# Solvent Co-Intercalation Reactions for Batteries and Beyond

**DOI:** 10.1021/acs.chemrev.4c00805

**Published:** 2025-03-15

**Authors:** Guillermo A. Ferrero, Gustav Åvall, Knut Janßen, Youhyun Son, Yuliia Kravets, Yanan Sun, Philipp Adelhelm

**Affiliations:** † Institut für Chemie, Humboldt-Universität zu Berlin, Brook-Taylor-Str. 2, 12489 Berlin, Germany; ‡ SEEL Swedish Electric Transport Laboratory, 423 73 Gothenburg, Sweden; § Joint Research Group *Operando* Battery Analysis (CE-GOBA), Helmholtz-Zentrum Berlin, Hahn-Meitner-Platz 1, 14109 Berlin, Germany

## Abstract

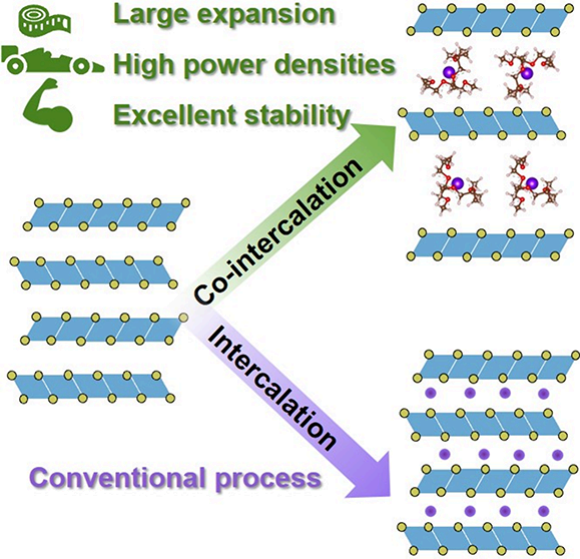

Solvent co-intercalation is a process in which ions and solvents jointly intercalate into a layered electrode material during battery charging/discharging. It typically leads to rapid electrode degradation, but new findings show that it can be highly reversible, lasting several thousand cycles. Solvent co-intercalation has two important characteristics: (1) the charge transfer resistance is minimized as stripping of the solvation shell is eliminated and (2) the fact that solvents become part of the electrode reaction provides another means of designing electrode materials. The concept of solvent co-intercalation is chemically very diverse, as a single electrode material can host different types and numbers of solvents and ions. It is likely that many undiscovered combinations of electrode materials, solvents, and ions capable of solvent co-intercalation reactions exist, offering a largely unexplored chemical space for new materials. Co-intercalation can expand the crystal lattice (>1 nm) to the extent that free solvents are present in the structure, forming a layered, “porous” material. This indicates that the concept has a much broader impact and relates to other research fields such as supercapacitors, layered nanostructures, and nanocatalysis. This Review covers the concept and current understanding of solvent co-intercalation reactions, characterization methods, advantages, limitations, and future research directions.

## 1 Introduction

Li-ion batteries (LIBs) can be considered as the most important energy storage technology developed in the last century. LIBs made their breakthrough in mobile electronics and are currently driving the transition to electric mobility (electric vehicles, EVs) and green energy (grid storage). Similarly, sodium-ion batteries (SIBs) are on the verge of entering the mass market. Despite their generally slightly lower energy content, SIBs can be made from more abundant elements and can be tailored to have other specific advantages, such as a lower price, a wider temperature window, or fast charging capability.^1−6^ SIBs are therefore seen as a novel technology complementary to LIBs.

**Figure 1:**
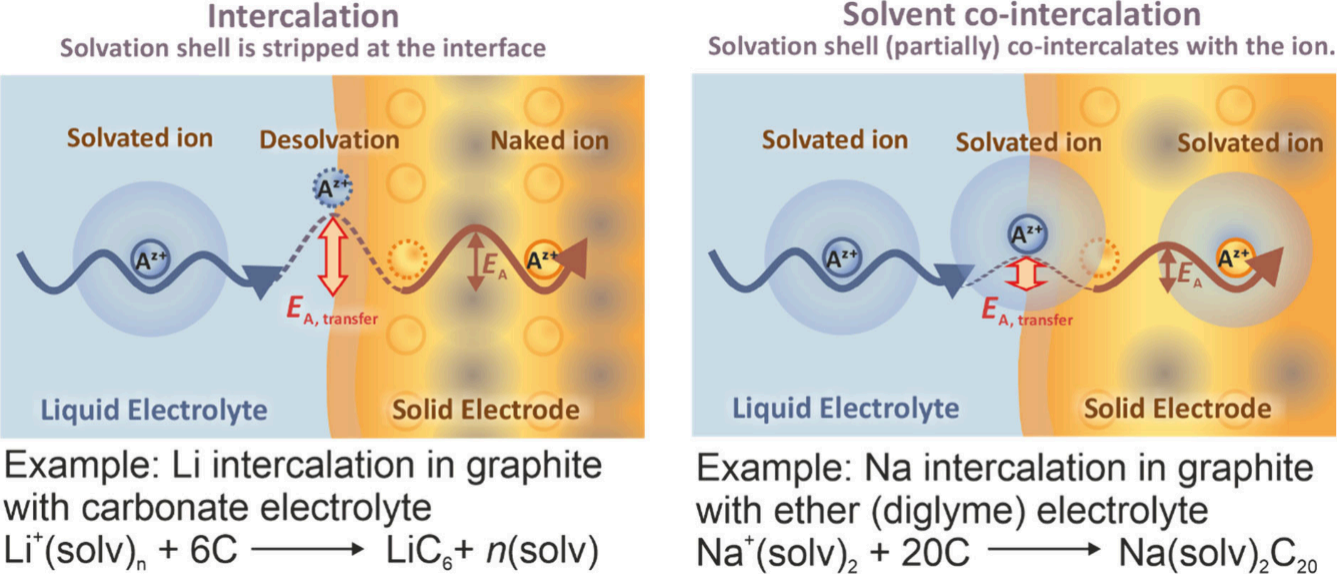
Sketch of the interface between the electrode and the electrolyte illustrating the difference between intercalation (left) and solvent co-intercalation (right). The arrows indicate diffusion of the solvated ion to the electrode, followed by charge transfer and diffusion of the ion in the electrode. For the conventional intercalation mechanism, the solvation shell is stripped during charge transfer, which requires a high activation barrier (*E*_A,transfer_). For graphite as the electrode, an SEI at the electrolyte/electrode interface ensures complete stripping (see also Figure 2). For solvent co-intercalation, solvents co-intercalate along with the ion into the electrode. The activation barrier for charge transfer becomes much smaller. (solv)_*n*_ represents the solvation shell of Li^+^, while *n*(solv) represents *n* free solvents. For Na^+^ and diglyme as the solvent, *n* = 2.

The concept of LIBs and SIBs relies on the reversible intercalation and deintercalation of Li^+^ (Na^+^) into electrode materials. The cell voltage is determined by the difference in the chemical potentials of Li (Na) in the electrodes, and ion transport between the electrodes is provided through a liquid electrolyte. Deintercalation of ions (A^+^) over the electrode/electrolyte interface is coupled to solvation, while the reverse process, i.e., desolvation combined with stripping of the solvation shell, occurs during intercalation (see Figure 1(left)). In a simplified way, the electrode reaction can be formulated as follows:

Deintercalation of ions from the electrode with solvation:
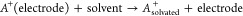
1

Intercalation of ions into the electrode with desolvation:
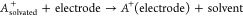
2

Hence, there is a strict separation of the chemical environment of the ions in the electrodes (crystal lattice) and the electrolyte solution (solvent molecules). The ionic conductivity of the electrolyte as well as the solvation/desolvation processes both impact the cell power and are detected as impedances in the system.^7^

It is important to realize that the solvation energies have a strong influence on the electrode redox potentials and can change their values by several hundred mV depending on the solvent.^8,9^ However, since desolvation and solvation occur simultaneously at both electrodes, there is no net effect on the cell voltage in a conventional alkali-ion battery.^2^ For this reason, solvent molecules are often omitted when describing electrode or cell reactions for LIBs and SIBs. In some cases, however, it is possible for solvents to enter the electrode host structure with the alkali ion through a process known as “solvent co-intercalation”. This breaks the otherwise common solvation-desolvation symmetry of metal-ion batteries.

Combined intercalation of ions and solvents:
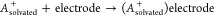
3

The difference between intercalation and co-intercalation is sketched in Figure 1 for graphite as the host electrode.

Historically, solvent co-intercalation was studied decades ago in regards to selected electrode reactions for primary battery applications as well as material synthesis.^10,11^ In the context of LIBs, the primary focus has been on the interplay between solvent co-intercalation and solid electrolyte interphase (SEI) formation, typically for graphite electrodes. In early studies, the co-intercalation of small amounts of solvents into the surface of the electrode material was identified as an important first step in the SEI formation process.^7,12−16^ Continuous solvent co-intercalation and subsequent electrolyte reduction, however, were found to cause graphite lattice degradation, especially in propylene carbonate-based electrolytes,^16,17^ leading to rapid cell failure and poor cycle life. It was therefore generally accepted that solvent co-intercalation is a highly detrimental process and an SEI is required to prevent any solvent to enter the graphite lattice.^18^

Approximately a decade ago, however, the research community became aware that controlled and continuous solvent co-intercalation into electrode materials can be a rapid and highly reversible reaction, rendering the process an intriguing avenue for battery research.^19,20^ The prototype reaction is the co-intercalation of Na^+^ and diglyme into graphite, leading to ternary graphite intercalation compounds (*t*-GICs).^19^

The fact that the reaction is both reversible and fast is somewhat surprising given that diffusion of a full solvation shell occurs rather than that of a small ion. However, in the absence of desolvation processes, solvent co-intercalation reduces the activation barrier for charge transfer across the electrolyte/electrode interface, which is typically dominated by the desolvation energy. This results in markedly reduced interfacial resistances and a decline in the activation energy, as shown for Li^+^ transfer.^21^ In addition, the solvation shell acts as an electrostatic shield to the ion, minimizing the electrostatic interaction between the Na^+^ and the negatively charged graphite lattice. For that reason, solvated Na^+^ can diffuse in graphite faster than bare Li^+^, resulting again in improved reaction kinetics.^22−24^

Solvent co-intercalation may also be a viable method for enabling reversible intercalation of multivalent ions such as Mg^2+^ or Ca^2+^.^25,26^ These ions are typically poorly mobile in electrode materials because of their high charge/radius ratio, which is an important bottleneck for the development of rechargeable multivalent batteries.^27^ The electrostatic shielding provided by the co-intercalated solvents can effectively increase the ion mobility if the lattice allows sufficient space.

More importantly, solvent co-intercalation may enable ion storage that would otherwise not be possible. The most illustrative example is the intercalation of Na^+^ into graphite, which is thermodynamically unfavorable for bare Na^+^ but possible by co-intercalation with ether molecules.^28,29^ The co-intercalation of solvents into electrode materials can therefore greatly expand the variety of electrode reactions currently known, and the topic has recently gained momentum.^30−34^ At this point, we emphasize that although many ternary intercalation compounds have been chemically prepared, this Review will only focus on electrochemically prepared compounds. Readers interested in chemically prepared ternary intercalation compounds are referred to the early work from Whittingham^35^ and Zabel and Solin.^11^

It is also important to realize that solvents co-intercalating into the electrode material will cause changes in the electrode potential and cell voltage based on their interaction with the materials. Unlike conventional LIBs and SIBs, where only Li^+^/Na^+^ interacts with the crystal structures of the electrode material, solvents now also interact, providing an additional means of altering the electrode properties. Electrode properties can therefore be tuned not only by changing their compositions and structures, as is well-known for layered oxides (NMC and NCA chemistry) and phosphates (LFP, LFMP) in Li-ion batteries,^36−38^ but also by co-intercalating specific solvents. As a result, this will cause the electrode to behave very differently than when the bare ion is intercalated.

The chemical diversity becomes even larger if multiple solvents co-intercalate at the same time or, in the case of different solvents, co-intercalate at the anode and cathode. For example, Zhang et al. and Escher et al. showed the combined co-intercalation of diglyme and ethylenediamine (EN) with Na^+^ in graphite.^39,40^ As both diglyme and EN can co-intercalate into graphite on their own, this approach seems straightforward. The use of certain solvents as promoters allows further enrichment of the compositions. Son et al. showed that diglyme can also act as such a promoter, i.e., diglyme enabled the co-intercalation of additional solvents [tetrahydrofuran (THF) and dioxolane (DOL)], which are unable to co-intercalate on their own.^41^ For both cases, quaternary graphite intercalation compounds (*q*-GICs) were formed, i.e., compounds consisting of reduced graphene layers between which sodium ions and two different types of solvents were sandwiched. The properties of these complex compounds are hardly known and explored to date.

This Review intends to give a critical overview on chances and challenges related to the concept of using solvent co-intercalation for electrode reactions in batteries. Intriguing results have been published in the past few years, and only a small number of possible reactions have been explored to date. Recently, the first co-intercalation battery (CoIB) has been demonstrated, which relies on solvent co-intercalation at both the negative (graphite) and positive (TiS_2_) electrodes of a Na-ion battery.^42^ At the same time, there is great need to better understand the boundary conditions for solvent co-intercalation as well as the behavior of co-intercalated solvent molecules during ion diffusion. Only when these aspects are understood will it be possible to fully explore the probably extremely diverse chemistry of co-intercalation reactions and assess their suitability for rechargeable batteries or other applications.

A conceptually similar approach of tuning properties of electrode materials is the use of molecules that can act as pillars to the layered structure or cause their delamination. In these cases, the molecules are typically chemically inserted with the aim to permanently change the structure.^43,44^ This is different from the solvent co-intercalation process discussed in this Review, which aims to be a reversible process that also occurs only during ion intercalation. Needless to say, however, there can be mixed cases.

## 2 Intercalation vs Co-Intercalation

**Figure 2:**
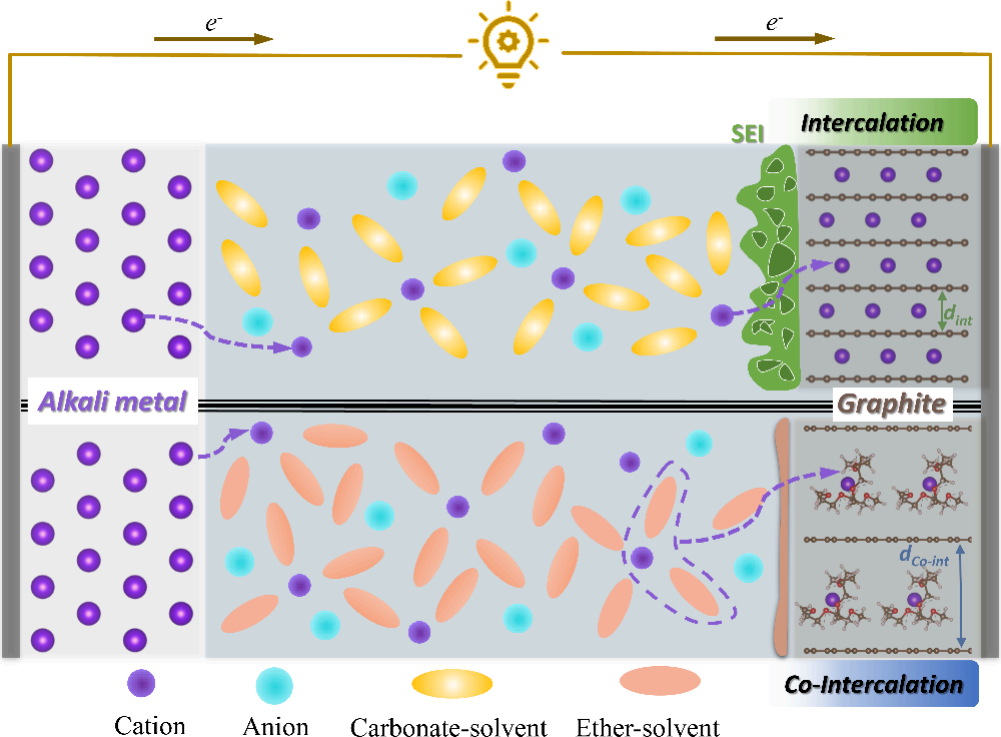
Sketch of electrochemical half cells with alkali metal electrodes (left) and graphite electrodes (right). For graphite electrodes, either intercalation or co-intercalation can occur depending on the electrolyte solvent. (Top) Intercalation mechanism with charge transfer through an SEI. The interlayer spacing *d*_int_ only increases by about 10% (Li intercalation). (Bottom) Combined intercalation of the ions and solvents. Depending on the solvent and the intercalating ion, the interlayer increases by more than 200% (values between 1 and 2.5 nm, i.e., micropore range, have been reported).^11^

The difference between intercalation and co-intercalation can be understood most easily, taking graphite as a representative example (see Figure 2). In today’s LIBs, graphite is the most frequently used anode material due to its cost-effectiveness and its favorable redox properties.^45^ The reductive intercalation of Li^+^ into the layered graphite structure occurs at potentials below about 0.25 V vs Li^+^/Li. The process involves the formation of binary graphite intercalation compounds (*b*-GICs) via several intermediates and ends once all interlayers are fully occupied (stage I has been formed). The composition of this fully lithiated graphite is LiC_6_, which corresponds to a theoretical capacity of 372 mAh g^–1^.^18,46,47^ The *b*-GIC forms according to the following electrode reaction:
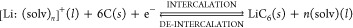
4where (solv)_*n*_ represents the solvation shell of Li^+^, while *n*(solv) represents *n* free solvents. As can be seen, the lithium cations are in a solvated state prior to intercalation. However, the electrolyte is unstable at the potential where ion intercalation in graphite occurs, leading to decomposition reactions and, therefore, formation of a solid electrolyte interphase (SEI) on the surface of the graphite during the first charge/discharge cycles. Because the SEI is a dense layer and electronically insulating, solvent co-intercalation and reductive electrolyte decomposition are eventually prevented. Only Li^+^ can pass through the SEI, finally enabling the desired reaction.^18^ In particular, carbonate-based electrolytes are used to form a SEI with favorable properties.^48^ A similar intercalation mechanism (although with a much larger graphite interlayer distance) is observed in the case of potassium with a specific capacity of 279 mAh g^–1^ to form KC_8_.^49,50^ In contrast, Na^+^ ions intercalate only to a very limited extent, and reported capacities from electrochemical measurements are well below 30 mAh g^–1^.^51−54^ This limited intercalation highlights the instability of sodium intercalation into graphite, especially when compared with the more stable intercalation of lithium and potassium. The reason for this behavior, which can be understood using DFT, is due to higher ionization energy and significant structural deformation caused by the larger sodium ions, leading to unfavorable stretching of C–C bonds as demonstrated by using theoretical calculations.^28^ Within the alkali metal series, Li rather than Na should be seen as the exception as a result of its unique binding energy characteristics and stronger van der Waals interactions.^28,29^

The lack of Na-rich *b*-GICs can be circumvented by using ethers instead of commonly used carbonates as electrolyte solvents. In this case, solvent molecules co-intercalate with Na^+^ to produce a ternary graphite intercalation compound (*t*-GIC) according to^19,20,55^
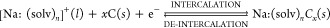
5

This process is called a *co-intercalation reaction* (or more precisely a *solvent co-intercalation reaction*, to emphasize that the solvent is the additional intercalating species). Note that the term co-intercalation is also used to describe the combined intercalation of two different cations into a host lattice, e.g., Mg^2+^ together with Li^+^ (more precisely *dual cation co-intercalation*).^56^ Co-intercalation may even be used to describe the intercalation of a charged complex such as [AlCl_4_]^−^, which is being studied for Al-ion batteries. These types of co-intercalation, however, are outside the scope of this Review.

The solvent co-intercalation reaction of Na^+^ with diglyme as the solvent was found to be surprisingly reversible, fast, and stable over several thousands of cycles.^19,57^ The co-intercalation mechanism is not restricted only to sodium and ethers and has been detected for other alkali metals such as lithium,^55,58^ potassium^55,59−62^ and, to a lower extent, the alkali earth metals calcium^63−65^ and magnesium.^25^ Most co-intercalation studies have used linear ethers (glymes) as solvents and reactions were studied by changing the type of glyme, the conductive salt, and the temperature.^8,53,66^ Next to linear ethers, however, co-intercalation is also found for amines^39,40,67^ and amides.^63,68^ Recently, cyclic ethers, dioxolane (DOL), DMSO and even water have been investigated.^41,69,70^ In addition, next to graphite,^8,31,39−41,53,55,58,63,66−70^ solvent co-intercalation has been also reported for few other host structures, namely dichalcogenides,^42,71−75^ Mxenes,^76−78^ polymers,^79^ titanates,^80^ and oxides.^81^

As can be deduced, the co-intercalation of a cation with a solvent molecule, in contrast to an intercalation reaction, results in a high volume expansion of the host structure, which in the case of graphite with Na^+^ and diglyme (2G) is in the range of 200%.^82,83^ Nevertheless, the electrode materials exhibited a high rate capability (up to 60 C) with high cycling stability and Coulomb efficiency.^8,19^ The absence of a desolvation step and an “SEI-free” interface are the reasons for this improved electrochemical performance. In contrast, the lower specific capacity achieved as well as the higher voltage plateau result in diminished energy densities compared with those of the conventional lithium intercalation mechanism. In the next section, an overview of the fundamentals of this reaction will be provided.

## 3 Fundamentals

### 3.1 The Reaction

In an ordinary LIB, taking the prototype cell chemistry (−)graphite|electrolyte|LiCoO_2_(+) as example, the half-cell reactions are given byPositive:
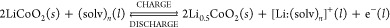
6Negative:
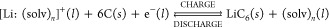
7Yielding the net reaction of the full cell:Net reaction:
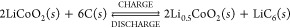
8

The energy released by the reaction is the difference in the Gibbs free energy between the reactants and products (Δ*G*). This value is linked to the cell voltage (*E*) by *E* = −Δ*G*/*zF*. As the solvent does not appear in the net reaction, the cell voltage is independent of the electrolyte solvent.

This changes when co-intercalation takes place. To illustrate this, we consider the cell (−)Na|electrolyte|graphite(+) as an example, with a linear ether as electrolyte solvent. The reactions are then as follows:Positive:
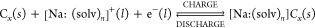
9Negative:
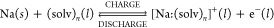
10Net reaction:
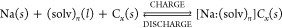
11

Comparing eqs 8 and 11, one can directly see that for the latter the solvent now becomes part of the net reaction, i.e., the solvent becomes an active material. This also means that the cell voltage changes depending on what solvent is being used. The electrolyte concentration can also influence the net reaction, and indeed many papers have noted that the reduction potential and the kinetics of the solvent co-intercalation reaction are sensitive to small changes in electrolyte composition, such as the salt concentration and type or number of solvents.^8,40,42,53,61,84^ Overall, the properties of an electrode and cell operating via a solvent co-intercalation mechanism are directly tunable by altering the electrolyte composition. This also means that a system displaying solvent co-intercalation can be used to electrochemically study the electrolyte, as the electrode potentials now depend on the electrolyte properties. Notably, the redox potential of the graphite electrode directly indicates the driving force for the co-intercalation process. For graphite, co-intercalation of Na^+^ and diglyme starts at potentials of around 1 V vs Na^+^/Na, which is higher than what is found for the intercalation of Li^+^.^19,55^ This means that there is a comparatively strong interaction between Na^+^, solvents, and the reduced graphite lattice, which clearly exceeds the energy penalty required to expand the graphite lattice. The calculation, however, of the redox potential is made more difficult compared to a normal intercalation process, as it requires exact knowledge of the conformation, position, and interaction of all components.

### 3.2 Effect on Host Structure

In ordinary ion intercalation, the expansion and deformation of the host structure are often small, and so is the energetic cost of the deformation. In the case of the co-intercalation of ions and solvents, however, the expansion is, in general, substantial. As the electrode material must accommodate a much larger intercalant, one of the most noticeable effects on the host structure is a large expansion of its lattice.^31,82^ Two of the most important techniques to identify and characterize solvent co-intercalation are thus X-ray diffraction (XRD), revealing the expansion of the crystal lattice, and electrochemical dilatometry (ECD), showing the macroscopic expansion of the entire electrode. These two techniques are complementary and typically show that the macroscopic expansion of the lattice is much smaller compared to the crystallographic expansion.^40^ This is because electrodes consist of particles and there is void space that can partially accommodate volume changes.^85^ XRD and ECD are reviewed in more detail in section 4.

The expansion of the material caused by co-intercalation is typically very large. For instance, graphite will undergo a huge expansion from its pristine 3.35 Å interlayer distance to an expanded one in the range of 11–13 Å.^53^ Larger values up to 25 Å for K(DMSO)_*y*_C_*z*_ have also been reported.^11^ At such large distances, the binding energy between the graphite sheets is practically zero.^86^ That is, the graphene sheets would delaminate at room temperature without the presence of the solvated ions.^42,71,72,87,88^ Thus, the solvated ions act as a glue, keeping the graphene sheets together. However, the stacking sequence changes from AB stacking in the pristine material to a mixture of AA and AB stacking with co-intercalation in a diglyme-based electrolyte.^86^ Co-intercalation can also cause the material to become amorphous, as observed in TiS_2_ or when propylene carbonate (PC) is co-intercalated into graphite,^42^ or it might alter the phase evolution of the electrode material.^89^ Again, these changes to the host material are best studied using a combination of XRD and ECD, the latter method having the additional advantage of being sensitive to changes in amorphous materials.

As the layered active material is generally expanded to such an extent that the layers are free from one another, the cost of expanding the lattice is the full binding energy between the layers. Hence, for this cost to be as low as possible, layered structures with quite weak interlayer binding energies are prime candidates as hosts for solvated ions. Moreover, the expanded materials’ reactivity is altered as generally the surface area of the electrode is greatly increased and the interior bulk of the material, normally isolated from the electrolyte, comes in direct contact with solvent molecules.

### 3.3 Role of the Solvent and the Electrolyte Concentration

**Figure 3:**
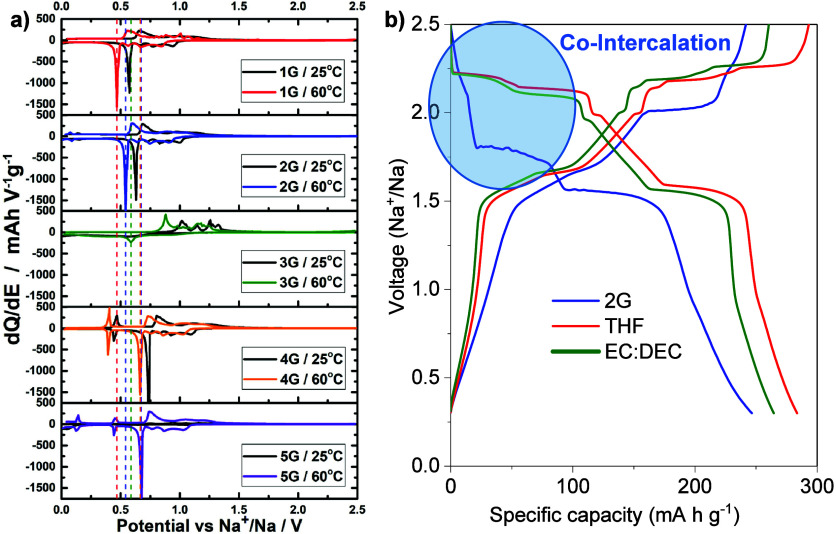
(a) Differential capacity plots for graphite electrodes with 1 M NaOTf for the different glyme solvents at 25 and 60 °C. Reprinted with permission from ref (8). Copyright 2018 American Chemical Society. (b) Galvanostatic sodiation and desodiation potential profiles for TiS_2_ electrodes with 1 M NaPF_6_ for different electrolyte solvents (2G, THF and EC:DEC), where co-intercalation occurs in the glyme-based electrolyte. Reproduced with permission from ref (42). Copyright 2022 The Authors.

In the case of a co-intercalation reaction, the type of solvent and the electrolyte concentration will directly impact the electrode properties. The type of solvent will directly affect the potential profile of the electrode, as the solvent will determine the free energy of solvation, the needed lattice expansion, and the solvation shell–host structure interaction. This effect is clearly seen in glymes with different chain lengths, where an increase in the potential occurs with the increase in size of the glyme (monoglyme (1G) to tetraglyme (4G)), shifting the solvation free energy (see Figure 3a).^8,20,53,84^ Generally, notable changes in the potential profile and capacity for the same electrode material using different electrolytes are the first signs that a co-intercalation reaction may occur. For instance, in the case of TiS_2_, it is evident that the reaction mechanism, along with the capacity and voltage, changes depending on solvent used (see Figure 3b).^42^ Furthermore, the co-intercalation in this host structure only happens at the beginning of sodiation and competes with an intercalation reaction.^42^

Solvent co-intercalation also leads to a depletion of solvents in the electrolyte solution, hence increasing its salt concentration.^86^ This effect can be quite pronounced, depending on the amount of electrolyte and active material in the system. As the solvation shells become less and less stable with increasing salt concentrations, this effect can lead to a system changing from a co-intercalation reaction to an intercalation reaction^61^ or in extreme cases to a drying-out of the cell.^86^ As will be discussed further below, changes in the electrolyte concentration are an important indicator for determining the number of solvent molecules that are co-intercalated per ion into the electrode.

### 3.4 Stoichiometry of Reaction

Already in the first papers published on reversible electrochemical solvent co-intercalation there are clear disagreements on the assumed number of glyme molecules being involved in the co-intercalation reactions.^19,83^ This was often only seen indirectly, as simulations of solvation shells inside the host structure contained either one or two diglyme molecules.^82,83^ However, few studies have tried to actually measure how many solvents are involved in the reaction. One possibility is to simply measure the weight change of the electrode, as was done as early as in the 1980s when co-intercalation was investigated for applications in primary batteries.^10,90,91^ The earliest experimental study reporting this for reversible solvent co-intercalation was published by Kim et al.,^83^ and they concluded that one diglyme molecule co-intercalated per Na^+^. However, NMR studies indicated that after desodiating the graphite, more than 25% of solvents remain inside.^92^ Mass measurement were carried out in several other studies.^63,83,84^ For Ca^2+^, it was shown that one calcium cation can be coordinated with four solvent molecules (DMAc). However, this mass measurement method has been used to report that one molecule of diglyme was intercalated.^72^

While mass measurements seem straightforward at first, they are quite complicated to conduct and nontrivial, as there are some intrinsic drawbacks to consider.^86^ Using an improved measuring protocol and combining results with other methods, we found that the number of co-intercalating solvents participating in the reaction depends on the state of charge and that the formation mechanism resembles more a pore forming and pore filling process rather than a continuous intercalation.^86^ Moreover, the results showed that both free solvents and solvents coordinating to Na^+^ are present inside the graphite structure, in line with NMR measurements.^93^ Overall, the co-intercalation process is much more complex than previously thought, and determining the correct number of solvents co-intercalating requires the use of several methods. The following section will summarize a number of methods that can be used to characterize solvent co-intercalation reactions.

## 4 Techniques to Detect and Characterize Solvent Co-Intercalation

In battery research, electrode materials and electrode reactions are studied with a large variety of analytical tools. Next to a number of electrochemical methods, microscopy (SEM, TEM), X-ray diffraction (XRD), and X-ray photoelectron spectroscopy (XPS) are among the most frequently used tools to study charge storage behavior and properties such as particle size and morphology, crystal phases, or SEI formation. Retrieving more detailed information and studying the dynamic behavior during cell cycling requires the use of more complex methods, including synchrotron radiation^94,95^ or neutron sources^96^ and operando/in situ experiments, for example. While all these methods are applicable to solvent co-intercalation reactions, the peculiar reaction mechanism requires (or allows) the use of additional methods and experiments. A particular challenge is to provide evidence that solvent co-intercalation instead of normal intercalation (or another storage mechanism) takes place.

Therefore, this section discusses which analytical tools can be used to study solvent co-intercalation reactions and how the experimental data compare between intercalation and co-intercalation.

### 4.1 Ex Situ, In Situ, and Operando Measurements

Analytical methods can be classified according to the manner in which the samples are analyzed, distinguishing them as ex situ, in situ, or operando. Ex situ experiments are easy to conduct, although they require stopping the electrochemical measurement to extract the electrode for analysis. Only selected samples, e.g., electrodes at specific states of charge (SOCs), can be studied. A major drawback is that the sample may easily change, e.g., by surface oxidation or because electrodes need to be dried and cleaned. In situ and operando techniques, where structural or chemical information can be obtained largely undisturbed and ideally during battery charging/discharging, are therefore preferred. The difference between in situ and operando measurements is often not very clear, and interpretations may also depend on the research discipline.^97−99^ Generally speaking, operando measurements are closest to the conditions that occur in a real battery. In operando experiments, data are typically continuously collected during charging/discharging. Examples include electrochemical dilatometry^100^ or microscopy.^101^ In situ measurements are close to this scenario, but typically the charging/discharging process is stopped until a measurement, e.g., recording of a diffractogram or spectrum, is completed. Both methods provide information that is otherwise hidden in ex situ experiments. For instance, ex situ XRD allowed the observation of the reversible expansion of the graphite lattice by co-intercalation,^51^ while operando experiments showed that staging occurs.^83^ Similarly, Raman spectroscopy has been used in both ex situ and in situ modes.^102^ Although in situ/operando methods are preferred over ex situ methods, a drawback of theirs is that, although they are used with the intention of getting close to the behavior in a real battery, they often require a special setup and sample holders that may not resemble a conventional battery and may affect the electrochemical properties. Therefore, results need to be interpreted carefully.

### 4.2 Electrochemical Methods

A variety of electrochemical methods are available that can be used to study solvent co-intercalation. An indication of solvent co-intercalation can already be seen from potential profiles that are recorded through charge/discharge experiments at constant current (galvanostatic cycling with potential limitation, GCPL). Co-intercalation is indicated in the case that different potential profiles are found for different electrolyte formulations, as discussed in the previous section. In the case of graphite, these differences are quite significant.

**Figure 4:**
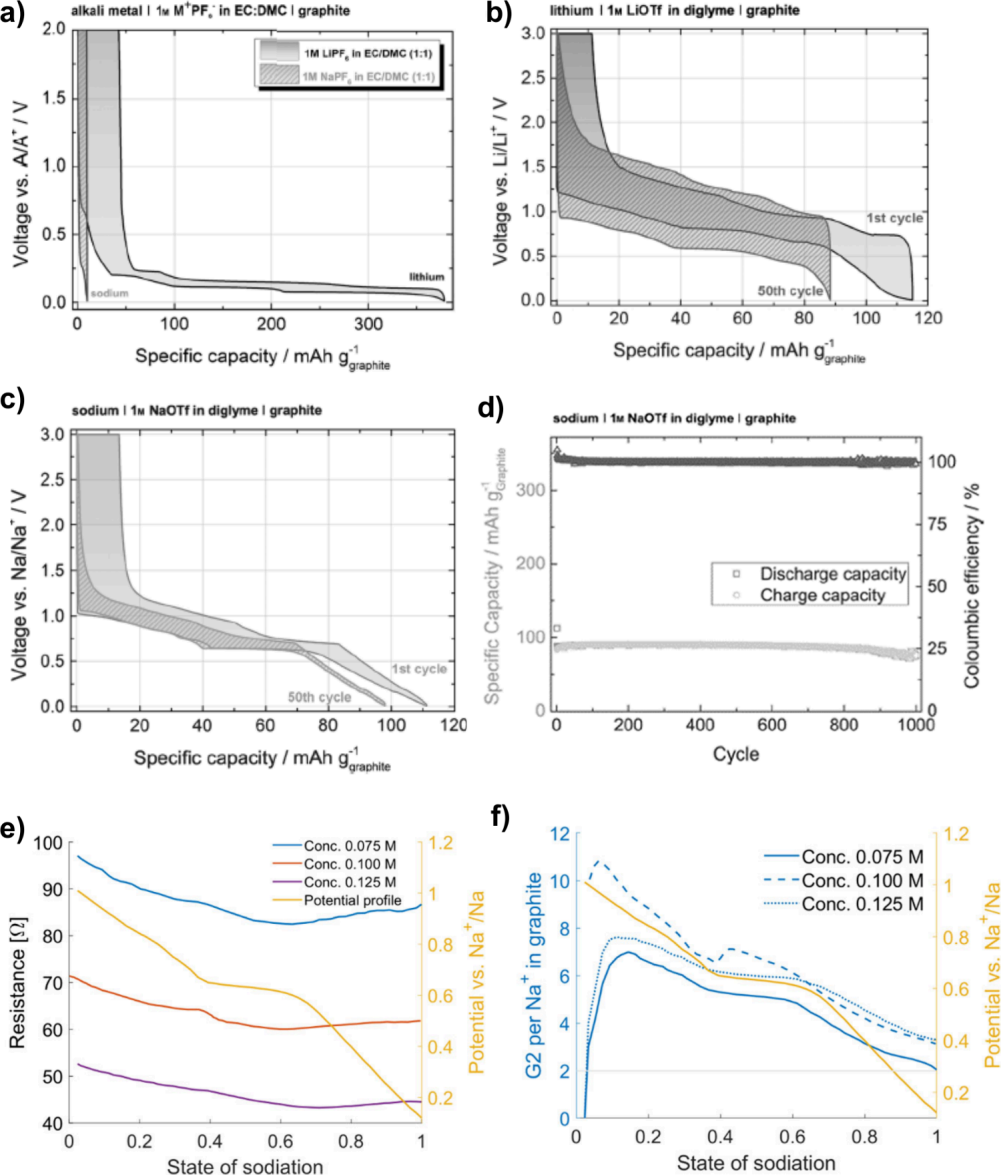
Voltage profiles (GCPL experiments) for charging/discharging Li|electrolyte|graphite and Na|electrolyte|graphite cells in different electrolytes: (a) 1 M M^+^(PF_6_)^−^ in EC/DMC and (b, c) 1 M M^+^(OTf)^−^ in diglyme. (d) Cycle stability and Coulomb efficiency of the Na|1 M NaOTf in diglyme|graphite cell over 1000 cycles at 0.1 C. Reproduced with permission from ref (51). Copyright 2014 The Authors. (e) Change in bulk electrolyte resistances of Na| NaPF_6_ in diglyme|graphite cells during sodiation for different salt concentrations (0.075 M, 0.1 and 0.125 M NaPF_6_) and (f) overall number of diglyme molecules being co-intercalated per Na^+^ (diglyme/Na^+^ ratio) during sodiation. Reproduced with permission from ref (86). Copyright 2023 The Authors.

In the seminal paper of Jache and Adelhelm, potential profiles of intercalation and co-intercalation reactions in graphite were compared using Li and Na cells and carbonate- and diglyme-based electrolytes.^51^ For Li intercalation and when using carbonate-based electrolytes, LiC_6_ is formed at potentials below 0.25 V vs Li^+^/Li, corresponding to a capacity of 372 mAh g^–1^ (see Figure 4a). However, when diglyme-based electrolytes are used, clear differences can be observed with a sloping region down to 0.55 V vs Li^+^/Li followed by a plateau (see Figure 4b). For Na, no activity is found for the carbonate-based electrolyte, which is in line with expectation as Na graphite intercalation compounds are thermodynamically unfavorable.^28,29^ However, when using a diglyme-based electrolyte, charge storage starts at about 1 V vs Na^+^/Na with a sloping potential and a plateau at about 0.65 V vs Na^+^/Na (see Figure 4c).

Despite the co-intercalation of large molecules into the graphene interlayer space, the overpotentials are relatively small in the case of Na, indicating efficient charge transfer and diffusion, the former being the result of the missing desolvation step. It is also of note that the co-intercalation reactions show a high Coulomb efficiency and therefore high capacity retention during long-term cycling (see Figure 4d). Reversibility over more than thousand cycles has been confirmed several times.^51,57,102^ Similar potential profiles were obtained for other metals such as potassium and calcium.^55,63^ In this regard, Kim et al. compared the electrochemical behavior of Li, Na and K in a glyme based-electrolyte with graphite. The results showed a comparable electrochemical performance with similar potential profiles and specific capacities at around 100 mAh g^–1^.^55^ However, the potential plateaus of the co-intercalation reactions varied between the alkali metals (Li < Na < K). The authors speculated that the expanded interlayer distance reduces the electrostatic repulsion, allowing larger guest ions to better stabilize the sodiated *t*-GIC and resulting in a higher potential.

The potential profile is also affected by the type of glyme solvent as shown in several papers. An increase in glyme length (from monoglyme to pentaglyme (5G)) generally leads to an increase in the plateau redox potential, which may be due to a more efficient electrostatic screening for longer glymes.^8,53,83^ In case of 3G and 5G, potential profiles show polarization at room temperature due to less ideal coordination (3G) and high viscosity (5G), which can be mitigated by increasing the temperature.^8^ The relevance of efficient coordination is also underlined by studies using ethers with poorly coordinating side groups (DPGDME, Butyl-2G), which also show polarization.^53^ An increase in temperature (60 °C) also enables co-intercalation of a crown ether (18c6) with a complex potential profile showing multiple steps.^8^ On a different note, the co-intercalation mechanism was independent of the type of electrolyte salt, except for cases where the measurement results were impacted by possible reactions of the salt with the counter/reference electrode. TFSI and FSI anions are especially problematic in this case.^66,103^

Cyclic voltammetry (CV) is another standard method used to characterize electrode reactions. It can be also conveniently applied to study co-intercalation reactions, and the impact of the solvent on the redox behavior of the electrode can be clearly determined.^53^ However, because in CV measurements the potential is controlled, currents can reach very high values that may damage the electrode.^51^ An alternative to CV is taking data from GCPL experiments and plotting d*Q*/d*E* vs *E*.^8,53,57,66,83,84^ In these diagrams, processes occurring at specific redox potentials can also be clearly identified.

Measurements of the cell potential as a function of temperature allow the determination of thermodynamic parameters such as the temperature coefficient (d*E*/d*T*) of the reaction, with *E* being the cell voltage (in equilibrium) and *T* being the temperature, or the entropy change (Δ*S*) of the reaction. For co-intercalation reactions, Goktas et al. estimated a temperature coefficient of about −2.55 ± 0.3 mV K^–1^ for a series of glymes, i.e. the potential plateau of the reaction decreases with increasing temperature.^8^ Using the temperature coefficient, the entropy change (Δ*S*) of the reaction can be determined using the following equation:
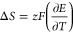
12

Values for d*E*/d*T* and Δ*S* are typically a function of the state of charge, which is often combined with the GCPL method, i.e., the cell is charged/discharged and the equilibrium potential is recorded by stopping the current in certain intervals. The method of determining the entropy change is also known as entropymetry.^104^ For example, this technique has been used for cathode electrodes to correlate the changes in entropy to the changes in the number of possible lattice configurations of the intercalated ions.^105^ More recently, Åvall et al. reported on the entropymetry technique to study the structural changes in the graphite electrode in the co-intercalation context.^86^ Since the measurements were performed in a half-cell configuration (Na|electrolyte|graphite), entropy changes should be dominated by the entropy changes in the electrolyte.^106^ The results obtained are in accordance to the staging observed in XRD; however, more work is needed to understand the possible use of this technique.

Overall, GCPL is an essential technique to detect and characterize solvent co-intercalation. Importantly, large changes in the potential profile due to changes in the electrolyte are a very strong indication that solvent co-intercalation is occurring. The main disadvantage of the technique is that it provides no direct data on how the active material is modified by the reaction, and therefore additional methods are required to gain a comprehensive understanding of the reaction.

#### 4.2.1 Impedance Spectroscopy

##### 4.2.1.1 General Considerations

Electrochemical impedance spectroscopy (EIS) can be a very powerful method to obtain detailed insights into co-intercalation reactions, especially with respect to the charge transfer mechanism. However, with typical EIS experiments being quite straightforward, meaningful interpretation of data for co-intercalation processes has so far been limited to a few cases. EIS is an electrochemical measurement technique where either the current response of a potential perturbation (PEIS) or the potential response of a current perturbation (GEIS) is measured.^107^

The interpretation of impedance data is often done by visualization of the data in a Nyquist plot, where the negative imaginary part and the real part of the impedance response are plotted against each other. Interpretation of the impedance data is done by fitting the data with a physically meaningful model (equivalent circuit model, ECM). The ECM consists of a series of elements such as capacitances and resistors representing, for example, bulk resistance, diffusion, or charge transfer processes. This way, quantitative data on the different processes causing cell impedance can be obtained. A frequently applied ECM is a variation of the Randles circuit with two RC circuits (visible as semicircles in the Nyquist-plot) for the migration of ions through the SEI and the charge transfer.^107^ For graphite in a sodium half-cell (diglyme with 1 M NaPF_6_), various results can be found in literature showing either one semicircle^108^ or two distinguishable semicircles.^109^ Thus, particular care must be taken when preparing the experiment and analyzing the data. In order to obtain good data, the results have to be gained in an experimental environment ensuring that the signal shown stems solely from the electrochemical processes of the working electrode or at least that the response of the counter electrode is known. The occurrence of an additional semicircle in a two-electrode setup might indicate influences from charge transfer or SEI resistance from the counter electrode.^110^ The challenge of interpreting impedance data from two-electrode cells can also be seen from results by Göktas et al., who studied the impedance of symmetrical (Na|electrolyte|Na) cells with glyme electrolytes containing different conductive salts.^82^ A three-electrode setup could mitigate these issues; however, the positioning of the reference electrode through the ion pathway of the electrons might add an additional semicircle. To circumvent these problems, when collecting data during impedance measurements for particular fast kinetic processes like the co-intercalation reaction, the most suitable setup would be to employ a microreference electrode such as that introduced by Linsenmann et al.^111^ Another suitable setup to effectively exclude any other contribution is a symmetrical cell setup, where two symmetrical working electrodes are used to build a counter-electrode-free two-electrode cell.^110^

##### 4.2.1.2 Electrochemical Impedance Behavior

Other EIS measurements show the same overall result: Lower overall impedance response of the glyme-based electrolyte system with co-intercalation occurring compared to typical intercalation systems with ester- and carbonate-based electrolytes.^20,109^

Though speculations have been made on the topic of film and/or charge transfer resistance contributions, the deconvolution of the single semicircle into two strongly overlapping signals by use of supporting measurements or evaluation methods (e.g., distribution of relaxation times (DRT)) has not yet been done for the particular case of the co-intercalation reaction. In recent studies, Wang et al. compared an ether-derived cell with a carbonate-based one in impedance measurements, obtaining two-semicircles for both configurations.^109^ They posited that between the two semicircles obtained through the Nyquist plots, the one in the higher frequency region can be attributed to an SEI evolution. According to this study, the impedance response of the ether-derived cell was significantly lower and thus exhibited beneficial kinetic properties despite SEI formation. Liu et al. showed that a high concentration of NaPF_6_ leads to the formation of NaF-rich interphases, causing a significant change in the impedance response.^108^ Furthermore, this NaF-rich interphase is believed to shut down the co-intercalation process and leads to lower discharge capacities. In addition, EIS has recently been used to determine the ratio of 2G molecules and Na^+^ during the co-intercalation reaction in graphite electrodes.^86^ In this regard, the co-intercalation reaction causes a variation in the amount of solvents in the bulk electrolyte in a half-cell configuration during sodiation and desodiation. Since the ionic resistance of the electrolyte, especially for low concentrations, is strongly dependent on the concentration, the change in bulk resistance and thus concentration can be tracked by in situ impedance measurements, as demonstrated by Åvall et al.^86^ By assessing these concentration changes, the average number of glymes that are intercalated together with the ion can be deduced (Figure 4e and f). In this regard, the impedance measurements corroborate the results of the mass change measurements that will be discussed in section 4.8.

When selecting an ECM for co-intercalation reactions, some unique properties related to the reaction should be considered. These can include the changing distance between electrodes due to the large expansion/shrinkage (“breathing”) of the electrode, which at the same time causes a variation in the pore size distribution of the electrode. Since the expansion of the interlayer distance between the graphene layers during co-intercalation increases drastically above 1 nm, the material could be considered microporous.^86,112^ This feature should also be taken into consideration when applying a model for the interpretation of impedance data. One model that accounts for phenomena associated with changes in porosity is the transmission line model (TLM), which considers the pore resistance encountered by ions as they migrate through cylindrical pores, leading to a more accurate representation of the impedance behavior in such systems.^113^

Overall, EIS can provide a lot of important information that is particularly useful to study solvent co-intercalation reactions. Notably, it can also be used to determine the number of molecules being co-intercalated into an electrode. On the other hand, interpretation of the EIS data remains challenging, and the data can be influenced by a multitude of factors, which somehow also limits its applicability and straightforward use.

#### 4.2.2 Galvanostatic Intermittent Titration Technique (GITT)

The GITT method is a very convenient way to study the apparent diffusion properties in electrochemical cells. Since the co-intercalation reactions show a high-rate capability, the method may therefore be particularly useful. GITT can provide quantitative data on ion diffusion coefficients (D) by introducing a series of current pulses, each followed by a relaxation time.^114^ Calculating the apparent diffusion coefficient from GITT measurements requires several boundary conditions and assumptions such as a dense and planar electrode, uniform current distribution, one-dimensional diffusion, and only minor volume changes during cycling.^114^ While these conditions are already not fulfilled for most battery electrodes, it becomes even more a challenge for electrode reactions with large volumes changes such as co-intercalation reactions.

Therefore, GITT measurements should be interpreted with caution, as the surface area (*S*) and molar volume (*V*_m_) of the electrode become a function of the state of charge, significantly impacting the result of the calculation done by the typically applied Weppner–Huggins approach (here simplified):^114^
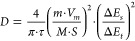
13τ is the pulse length, *m* is the mass of the active material, *M* is the molar mass, Δ*E*_S_ is the change of the voltage during the waiting time, Δ*E*_t_ is the total transient voltage shift during the time when the current is applied.

Nevertheless, because of its ease of application, the method can provide very useful information. For example, Li et al. compared the diffusion properties of solvent co-intercalation and intercalation reactions of potassium in graphite.^115^ While for co-intercalation the apparent diffusion coefficient appeared to be close to 10^–9^ cm^2^ s^–1^, intercalation led to values 2 orders of magnitude lower (10^–11^ cm^2^ s^–1^). Similar values for co-intercalation (10^–9^ – 10^–8^ cm^2^ s^–1^) were found by Escher et al. for the co-intercalation of sodium with ether-based electrolytes in various graphites.^22^ It is noteworthy that for co-intercalation reactions an asymmetric behavior in diffusion is observed between charging and discharging.^22,115,116^ Thus, during sodiation, a constant diffusion coefficient is observed that decreases significantly near the plateau at 0.625 V vs Na^+^/Na,^25^ which is likely due to the last stage formation. Interestingly, the decrease of the diffusion coefficient calculated is more pronounced during sodiation compared to the desodiation, indicating that solvated ion extraction is faster and the diffusion less hindered. Nevertheless, this plateau region might also be prone to errors, as fewer potential changes occur compared to stoichiometric variations, leading to a low difference in potential upon charging (Δ*E*_t_) in the typically applied simplified Weppner–Huggins approach.

Overall, GITT measurements have the advantage of being easy to perform. They allow changes in the diffusion properties of an electrode as a function of the state of charge to be easily identified, as well as trends within a series of samples. However, because the equation typically used requires many simplifications, quantitative evaluation remains a challenge, and the method does not provide direct information about the charge storage mechanism.

### 4.3 X-ray Diffraction (XRD)

**Figure 5:**
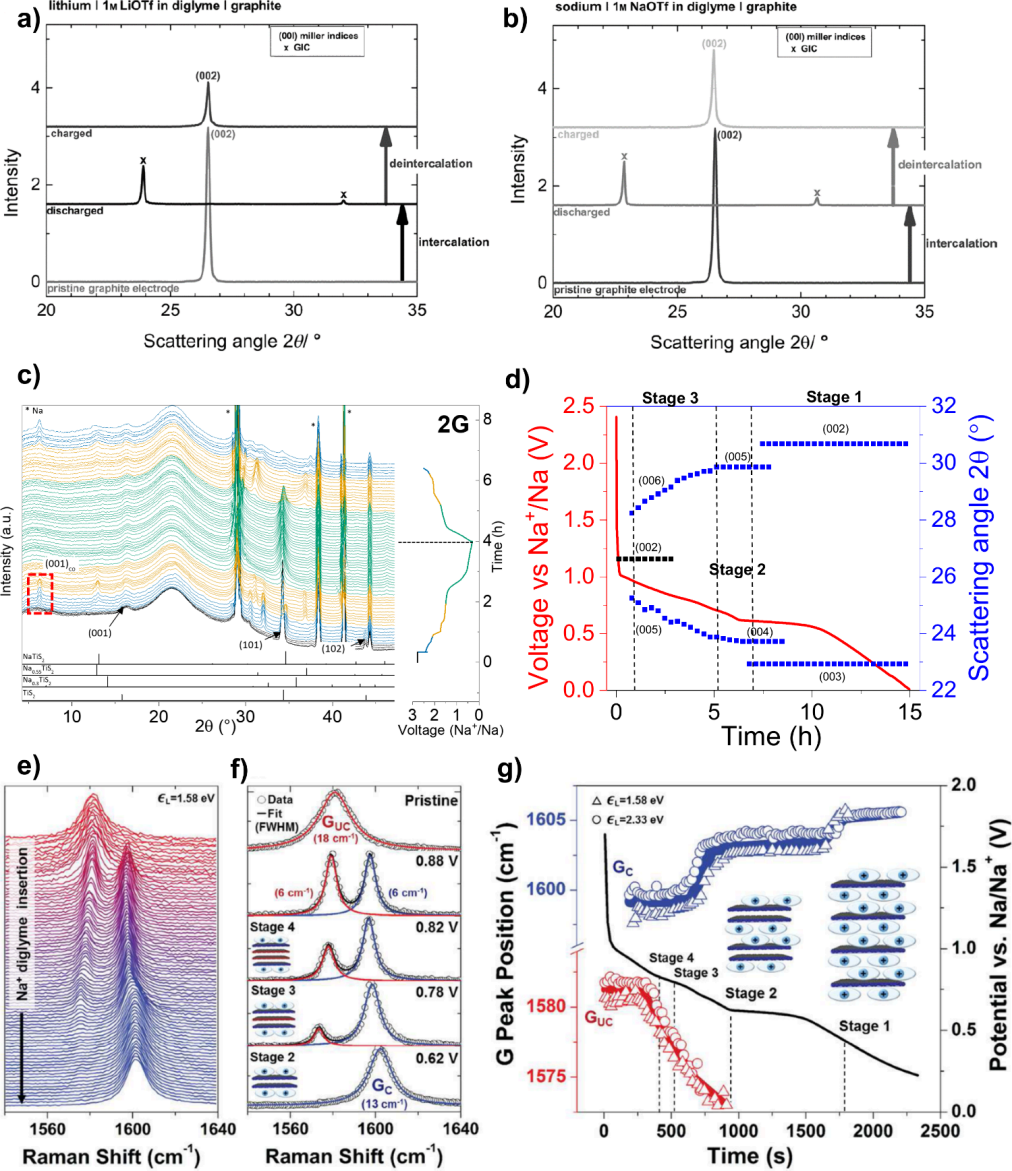
(a, b) X-ray diffraction patterns of the graphite electrodes in their pristine state, after electrochemical intercalation of solvated Li^+^/Na^+^ (discharged to 0.01 V), and after subsequent deintercalation. Reproduced with permission from ref (19). (c) Voltage profiles at 100 mA g^–1^ with the corresponding operando diffraction patterns of the TiS_2_ electrodes. Reproduced with permission from ref (42). (d) Evolution of the XRD peak positions of the graphite electrodes and the corresponding staging behavior. (e) In situ Raman spectra (normalized) of few-layered graphene showing the highly ordered staging reaction as measured using a 1.58 eV laser. (f) Selected spectra and Lorentzian fits of GC (blue line) and GUC (red line) bands. (g) Tracking of the positions of the Raman G peak components (GC shown in blue and GUC shown in red) measured in situ with the 1.58 eV laser (triangles) and the 2.33 eV laser (circles) during the electrochemical intercalation reaction, with the corresponding Galvanostatic discharge (∼0.2 A g^–1^) profile shown with respect to the right *y*-axis (black line). Reprinted with permission from ref (102). Copyright 2016 American Chemical Society. Panels (c–g) used 1 M NaPF_6_.

X-ray diffraction is probably the most commonly used method to detect solvent co-intercalation. This is because the intercalation of solvated ions into the layered host structure typically leads to a large expansion of the interlayer space, causing significant changes in the XRD pattern. Hence, XRD has frequently been employed for characterizing a variety of chemically prepared solvent-containing layered compounds based on graphite^117−123^ and TiS_2._^87^ The first XRD evidence of reversible solvent co-intercalation in graphite was provided by Jache et al. and Kim et al. for the co-intercalation of Na and diglyme in graphite.^19,20^ Once graphite is sodiated, the (002) peak located at 2θ = 26.5° splits into two peaks at around 23° and 29°, going into a different staging mechanism. These results are summarized in Figure 5a and b) for both Li and Na co-intercalation. At the same time and since the beginning of the main voltage plateau, an additional peak appeared at around 15°. Based on Bragg’s law, this corresponds to a volume expansion as large as 250%.^53^ After desodiation, the (002) peak of graphite reappears, proving the reversibility. In a similar way, the co-intercalation of numerous other solvents (different glymes,^53,124^ carbonates,^125^ and amines^39,40,67^) when using other cations^55,126^ was studied by XRD.

Recently, it has been also demonstrated that co-intercalation of diglyme can also be observed for TiS_2_ with the shifting of the (001) feature from 15.5° to 6.1° (see Figure 5c).^42,72^ In an earlier work, irreversible co-intercalation of PC with lithium was also reported.^88^ This indicates that while graphite remains the most studied system, also other compounds can show co-intercalation, yet with also varying degrees of reversibility.

It is worth noting that the co-intercalated materials are very reactive, so ex situ XRD measurements require airtight sample holders. More desirable are in situ (or operando) experiments (with an example shown in Figure 5d), which are already reported in the literature.^20^ As stated before, it is possible to follow the evolution of the sodiation based on the XRD peak position over time. Staging begins from the (002) graphite peak splitting into two, (005) and (006), peaks at the beginning of sodiation. During stage 2, the (004) and (005) peaks appeared until stage 1 formed at the main voltage plateau with the (002) and (003) peaks. From these positions and based on Bragg’s law, it is also possible to calculate the distances repeated between layers for stages 3, 2, and 1, around 18.83, 14.99, and 11.62 Å respectively. However, these are the repeated distances between the layers filled with the co-intercalated sodium ions. Hence, for example, the distance of stage 2 includes the distance of 2 graphite sheets with 1 sodium layer between them combined with the distance of 1 empty graphite sheet. Similarly, the distance for stage 3 includes 2 graphite sheets with one sodium layer and the distance of 2 empty graphite sheets. As related to the evolution of distances between two full graphite sheets, once sodiation starts, the distance increases until the formation of stage 2, after which it remains constant during the rest of the process. These results correlate perfectly with electrochemical dilatometry experiments, where the majority of the expansion occurs until stage 1 formation (see section 4.5). These findings indicate that it is possible not only to utilize XRD to determine changes in the crystalline structure and detect the formation of characteristic ternary intercalation compounds and their reversibility but also to investigate variation of the lattice parameters. Interestingly, as shown by Kim et al., the structural evolution and the staging behavior of Li, Na, and K in graphite with diglyme are similar, which is indicative of the minimal interaction between the alkali ions and graphite that are weakened by solvating molecules.^55^ At this point, it is worth mentioning that, as already reported, a proper interpretation of the data is of highly important to avoid wrong labeling of the peaks. Thus, as already observed in the case of TiS_2_, an observation in a narrow angular value can lead to an overlook of additional peaks that can explain a much higher lattice expansion (see Figure 5c).^42^

Finally, small-angle X-ray scattering (SAXS) is another method that appears promising for the operando characterization of solvent co-intercalation reactions.^127^ In this regard, the authors suggested that solvated Na^+^ was found to be stored in the grain boundary cavities and in mesopores which, in combination with the pseudocapacitance behavior, led to a coadsorptive sodium storage mechanism.^127^ The material used, however, was synthesized from cotton fiber that exhibited a combination of storage mechanisms and thus the exact effect or detection of the co-intercalation species cannot be easily assessed. Nevertheless, SAXS may become a very useful tool to study interlayer expansion and/or pore filling of solvent co-intercalation reactions, complementing the more commonly used X-ray diffraction experiments.

To conclude, XRD is the most important bulk technique for confirming the occurrence of co-intercalation and for identifying intermediate phases such as staging. Large lattice expansions, although not exclusive to solvent co-intercalation reactions, are a clear indication of that solvent co-intercalation takes place. XRD, together with GCPL, are likely the most convincing methods to prove solvent co-intercalation. In fact, if no large volume expansion is detected, solvent co-intercalation can be ruled out (unless the material in question already has very large open spaces in its pristine crystal structure). The main advantage of XRD is that it provides bulk information and quantitative information about the crystal structure. Despite the advantages, there are also a few disadvantages to using XRD. First, XRD becomes difficult to use when buckling/amorphization of the layered structure occurs, as in the case of TiS_2_.^42^ Second, compared to GCPL, XRD is typically applied ex situ or, if applied in operando mode, only one or at most a few cycles are studied, which somewhat limits the information on the long-term behavior of the reaction. Finally, the authors emphasize the importance of looking into the often neglected low angle region of the diffractograms, as this is where large expansions are most easily detected.

### 4.4 Raman Spectroscopy

Raman spectroscopy is a nondestructive measurement technique that can provide information about the structure and chemistry of electrode materials. In particular, it has been widely used to characterize carbon materials, especially graphitic materials.^128^ When the spectra measured for these compounds are analyzed, several characteristic features can be observed. The G-band peak appears at 1582 cm^–1^ and is an indication of the sp^2^ carbon network. The D-band and D′-band (at 1350 and 1620 cm^–1^, respectively) represent structural defects and therefore are not visible for pure graphite.^129^ Kim et al. detected changes to the structural order of graphite when sodiated with diglyme by using Raman spectroscopy.^130^ Once sodiated, an intensity increase of the D- and D′-bands was observed, since the material became less ordered and more defects or distortions appeared in the graphitic structure. After desodiation, the graphite structure returned to its original crystalline order, as confirmed by the disappearance of these bands. This behavior was also observed during the co-intercalation reaction in the case of other cations.^59,64,131^

In situ Raman spectroscopy provides a much clearer picture of the staging behavior, as can be observed in Figure 5e and f.^102^ Cohn et al. first observed the splitting of the G-band into two, which is characteristic of staging reactions in graphite (Figure 5g). The band at 1580 cm^–1^ represents the G-mode of an uncharged graphene layer (G_UC_), and the one at 1600 cm^–1^ corresponds to the G-mode of charged graphene (G_C_). Emergence of the G_C_ peak at 1600 cm^–1^ indicates the beginning of the staging process. As the intercalation reaction continues, the intensity of this peak increases, while the initial G-band (G_UC_) at 1580 cm^–1^ disappears, representing the absence of uncharged graphene layers (at stage 2). Based on their experiments, changes to the G-band represent the main route to analyzing evolution of the co-intercalation reaction. On the contrary, when the mechanism is a common intercalation, such as with lithium in carbonates, there is broadening and splitting of the G-band (at stage 2 or more), followed by a gradual loss of intensity when the intercalation continues.^132^

Following these findings, it can be concluded that Raman spectroscopy is very suitable for studying co-intercalation reactions. Care must be taken, however, as the black graphite electrodes absorb a significant share of the laser intensity and therefore might be damaged during the experiments. It is worth noting that Raman spectroscopy is also frequently used to study the solvation of ions in electrolyte solutions, as shown by, for example, Kondo et al., Xu et al., and Yamada et al.,^84,133,134^ which eventually may help improve the understand of the structure of co-intercalated solvated ions.

### 4.5 Electrochemical Dilatometry (ECD)

Operando electrochemical dilatometry (ECD) is a technique that allows the measurement of the thickness change of an electrode during charging/discharging (“electrode breathing”). ECD is typically applied during a GCPL experiment. The technique has been used in electrochemical experiments already since the 1970s^135^ and has the advantages of having a wide measurement range (nm to mm) depending on the conditions of the setup. Sometimes, the method is also referred to as the “in situ” method. However, as the method provides continuous data during battery charging/discharging, it is suggested that it be classified as an “operando” method. For a long time, however, the widespread use of ECD was limited by the lack of commercial devices. With commercial devices now being available, the method is receiving increasing attention, as it can provide, in addition to existing methods, quite complementary information on the dynamic electrode behavior of battery and even supercapacitor electrodes. The use of ECD in battery and supercapacitor research has been recently summarized.^85^

**Figure 6:**
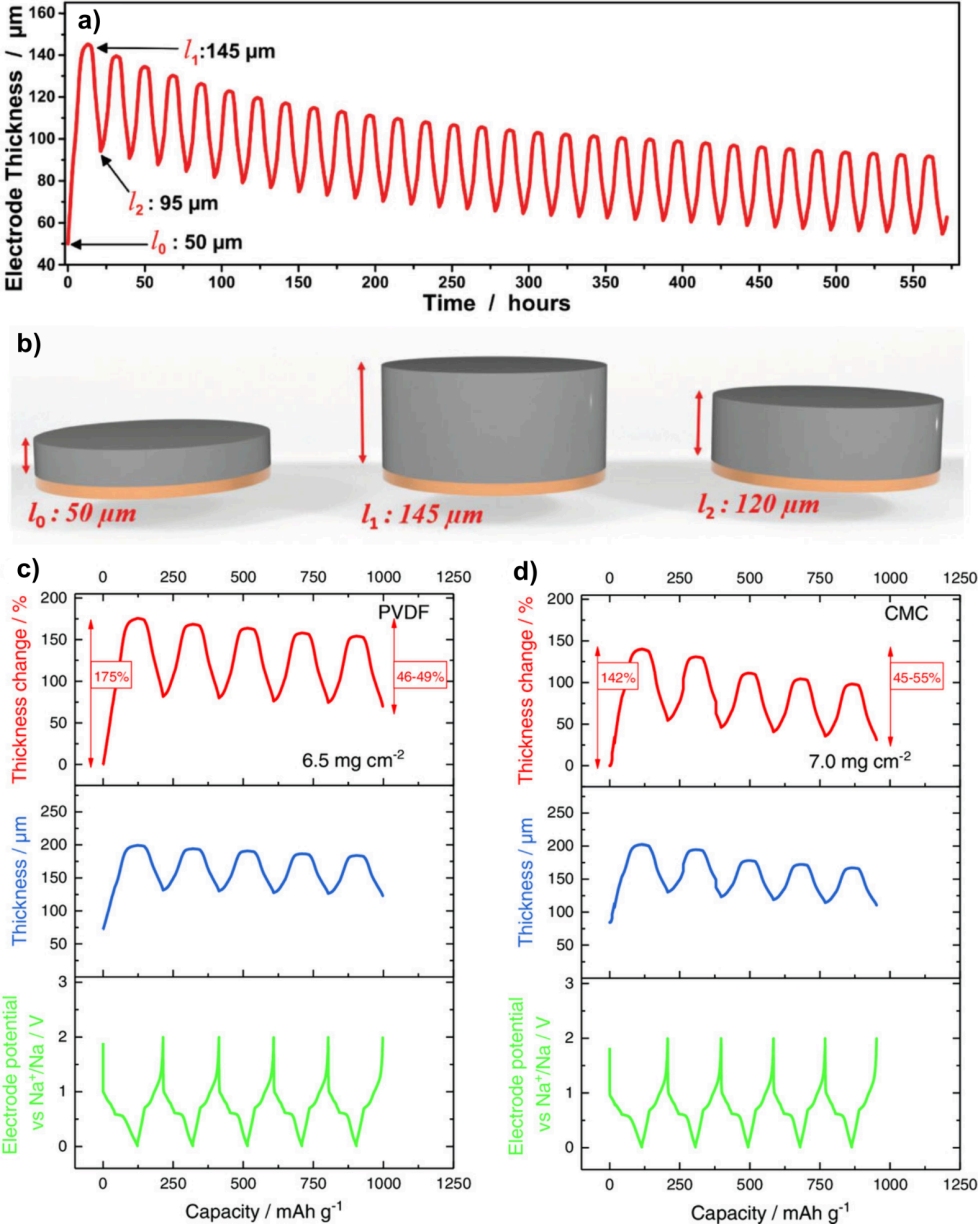
Operando electrochemical dilatometry (ECD). (a) Electrode thickness during cycling and (b) sketch of the dimensional changes of the electrode during the first cycle. Reproduced with permission from ref (82). Operando electrochemical dilatometry measurements of graphite electrode with two different binders (c) PVDF and (d) CMC. Reproduced with permission from ref (40). All the measurements were made in a three-electrode setup with graphite as the working electrode, sodium as the counter and reference electrodes, and 1 M NaPF_6_ 2G as the electrolyte.

Goktas et al. have examined the reversible sodium co-intercalation into graphite electrodes with ether-based electrolytes by ECD.^82^ The graphite electrode experiences large volume changes (expansion and shrinkage, “breathing”) during desodiation (70–100%) and shows no significant signs of hindrances to reaction reversibility (see Figure 6a and b). Additionally, these studies revealed that during the reaction staging the electrode thickness increases the most, while below the main plateau the expansion is much less pronounced. Afterward, Escher et al. reported a correlation based on data obtained through operando ECD between the type of binder and volume changes of graphite electrodes in SIBs. Figure 6c and d shows that CMC reduces the thickness change down to 142% during the first sodiation compared to PVDF (175%), after which both electrodes demonstrate a similar breathing effect. Furthermore, adding a cosolvent, in this case ethylenediamine (EN), had a strong effect on reducing the breathing of graphite electrodes during cycling (175% of expansion EN-free and 100% of expansion with EN).^40^ This technique has not been restricted to graphite electrodes. Thus, solvent co-intercalation in the TiS_2_ electrode with diglyme electrolyte in comparison with carbonate (EC:DEC) and THF electrolytes was investigated by Ferrero et al.^42^ Previous XRD data indicated the existence of solvent co-intercalation with a diglyme electrolyte at high voltages. In this work, the ECD results were in good agreement with the XRD data. A thickness expansion of 39% was observed with the diglyme electrolyte during the first sodiation compared to a 13% expansion in the case of THF.

Overall, ECD as a technique is very complementary to XRD and is particularly useful for co-intercalation reactions due to the large breathing that is typically observed. Compared to XRD, however, ECD is also sensitive to changes in amorphous materials, and measurements can also be conducted over many consecutive cycles. The method is also comparably affordable. On the negative, one has to be aware that ECD probes the free expansion of the electrode, which is different from a real battery where the cell stack is confined. When studying very small thickness changes, also the drift of the data over days/weeks must be considered.

### 4.6 Microscopy Methods

**Figure 7:**
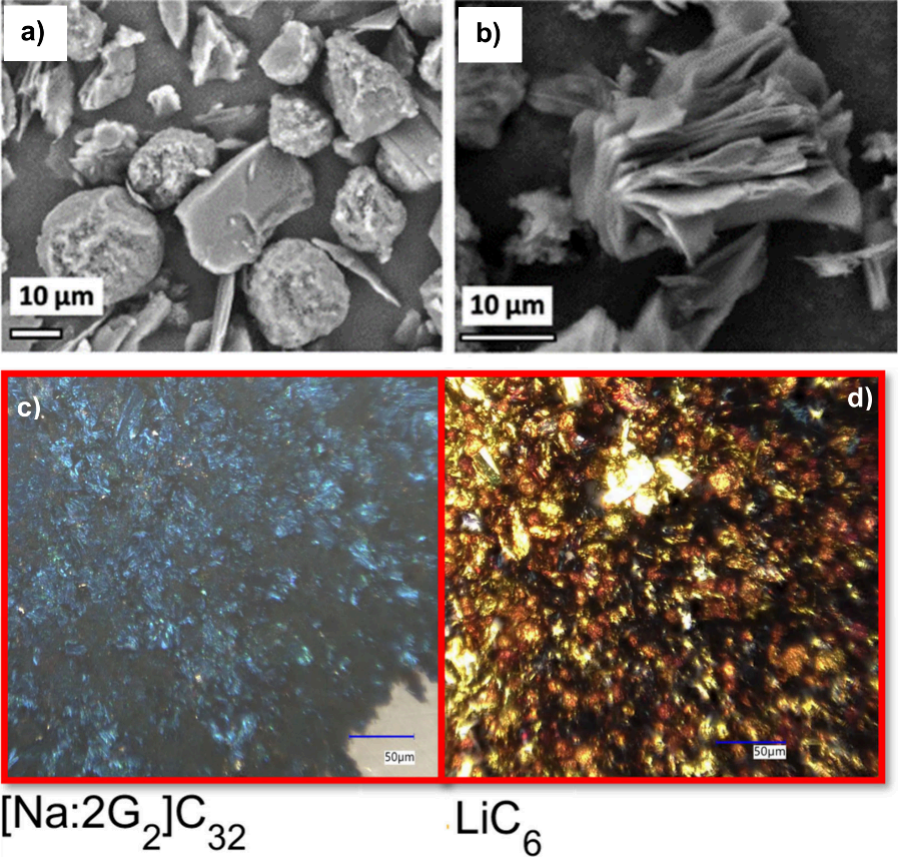
SEM pictures of (a) the pristine graphite particles and (b) graphite particles after solvent co-intercalation (second cycle, desodiated state). Reproduced with permission from ref (82). Microscopy pictures of the graphite at (c) full sodiation and (d) full lithiation. Reproduced with permission.^86^.

Microscopy methods are well suited to study electrode reactions, although on a regular basis the methods are typically applied ex situ. When applying scanning electron microscopy (SEM) or transmission electron microscopy (TEM), the sample preparation and the sample transfer often consist of several steps, which eventually may strongly influence the results. Washing of the electrodes as well as the air-sensitivity of many of the samples are the most common concerns when using these methods. On the other hand, the methods have been found to be very useful in studying co-intercalation reactions. Goktas et al. compared the morphology of graphite particles before and after solvent co-intercalation and found clear evidence for particle cracking and exfoliation.^82^ This is undesired, but it is important to note that the exfoliated particles remained crystalline graphite. Delamination, i.e., separation of the graphenes along with a loss of crystallinity, does not take place. This behavior is likely the cause for the long cycle life of the electrodes (see Figure 7a and b).^82^ SEM also confirmed the results from operando ECD, showing the change in the electrode thickness before and after cycling. Although there have been reports on the use of in situ and operando SEM for other battery mechanisms and configurations,^136^ this technique has not yet been used for co-intercalation investigation purposes.

TEM has already been used to study intercalation processes,^137,138^ although most commonly for detecting the SEI formation.^58,109^ TEM can be used to compared the interlayer spacing between graphene layers.^130^ Wang et al. used TEM to compare the local structure of graphite for different intercalation reactions.^139^ TEM images of potassiated electrodes showed distortions in the graphite structure when using EC/DMC as the solvent, while with ether-based electrolytes the graphite retained a relatively ordered structure. On the other hand, TEM studies to find surface films on graphite particles after a co-intercalation/de-co-intercalation cycle were done by Goktas et al. (using graphite powder without binder to exclude artifacts).^82^ The measurements did not show any indication of an SEI, which is why the authors proposed that the graphite surface is “SEI-free”. Besides the sample preparation, potential beam damage as well as the limited probed area are further limitations of TEM. Some of these challenges can be partially mitigated by cryo-TEM, which was recently employed for SEI detection.^140^ Use of an operando TEM configuration can also help with avoiding these difficulties.^141^

Recently, optical microscopy was used in operando mode to study co-intercalation reactions.^86^ The measurements over cycling were well in line with ECD measurements showing large breathing of the electrode during cell cycling. Optical microscopy is also well-suited to follow the staging during charging/discharging. While graphite turns golden when being lithiated to its full capacity (372 mAh g^–1^), a blue coloration is found for the formation of the *t*-GICs with Na and diglyme (about 110 mAh g^–1^), as can be seen in Figure 7c and d.^86,102^

**Figure 8:**
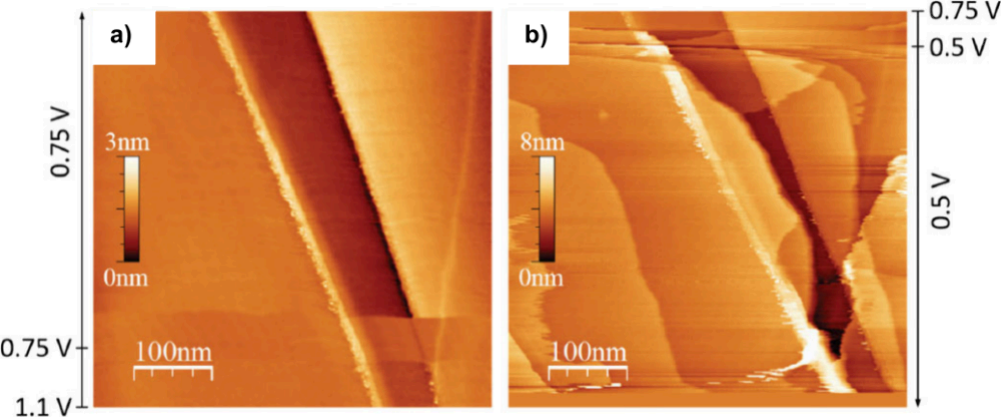
Operando EC-STM images at 5 mV s^–1^ cyclic voltammetry of highly oriented pyrolytic graphite in 1 M NaClO_4_ in triglyme when sodiation (a) from pristine graphite to 0.75 V vs Na^+^/Na and (b) from 0.75 V vs Na^+^/Na to 0.5 V vs Na^+^/Na. Reproduced with permission from ref (124).

There are further microscopy measurement techniques that can be used for detection of the co-intercalation process. Scanning probe microscopy (SPM) is commonly used to study surfaces with an electrically charged probe. Among the most known probe microscopy techniques, atomic force microscopy (AFM) is a type of SPM that operates at the nanoscale. Song et al. used AFM to investigate the interaction of diglyme (PC + diglyme) electrolytes with a graphite anode in lithium cells.^131^ Thus, by observing the electrode at different potentials, swelling effects caused by the co-intercalation reaction of solvated lithium ions were detected. Similarly, Song et al. identified the co-intercalation of solvated lithium ions into graphite during SEI formation in the first cycle via the detection of unchanged blister structures within the graphite layers (with the corresponding layer expansion), demonstrating the stability of these compounds.^142^ Scanning tunneling microscopy (STM) is another type of probe microscopy used to analyze electrode surface details. STM captures the surface image via quantum tunneling at the atomic scale by using a sharpened conducting tip, thus allowing the quantification of local topographical changes. Contrary to AFM, STM can be used only for imaging conductive or semiconductive surfaces. Due to this, AFM can be utilized to determine solvent co-intercalation, as Seidl et al. proved for several *t*-GIC (Na/glymes) and measuring lattices of single graphene sheets.^124^ In this case, highly oriented pyrolytic graphite was needed in order to image the electrochemical process. The lattice expansion of graphite was detected through the presence of horizontal lines, demonstrating that intercalation and expansion occur at ∼0.5 V vs Na^+^/Na as observed in Figure 8. This technique also allows a quantification of the lattice expansion by measuring the height profiles.^124^

### 4.7 Nuclear Magnetic Resonance (NMR)

Nuclear magnetic resonance (NMR) is a spectroscopy technique where the interactions of the nuclear magnetic moment with an electromagnetic field in the radio frequency are observed when a magnetic field is applied.^143^ In this sense, the nuclei resonate at a specific frequency, determined by their chemical environment, which can be measured and converted into an NMR spectrum. The local magnetic field of the nucleus is influenced by several parameters, such as the interaction between molecules or different molecular configurations. Therefore, NMR can provide relevant information about the chemical structure through the different chemical shifts of materials and their interfaces. Literature on the use of NMR has significantly increased over the last years, with investigations focusing on surface coatings, characterization of electrodes, and SEI formation and analysis.^144^ For a more comprehensive review of NMR as an analytical tool, the reader is referred to specialized literature, in particular the review focused on the employment of NMR for battery research by Pecher et al.^144^ As previously discussed for other measurement techniques, the feasibility of using NMR for the detection and characterization of solvated intercalation compounds is examined in this section.

The use of NMR for the characterization of graphite intercalation compounds has already been reported for binary^145^ and ternary intercalations compounds for different metal–(NH_3_)–graphite compounds^146^ and for amine–graphite and polyamine–graphite intercalation compounds.^120,123^ In those works, static and magic angle spinning (MAS) experiments were used for ^13^C and ^7^Li. In addition, Gotoh et al. proved the existence of ternary graphite intercalation compounds (Na/diglyme) with a given stoichiometry of C_22—26_(2G)_1.8–2.2_Na_1.0_.^147^ However, those materials were not synthesized electrochemically.

**Figure 9:**
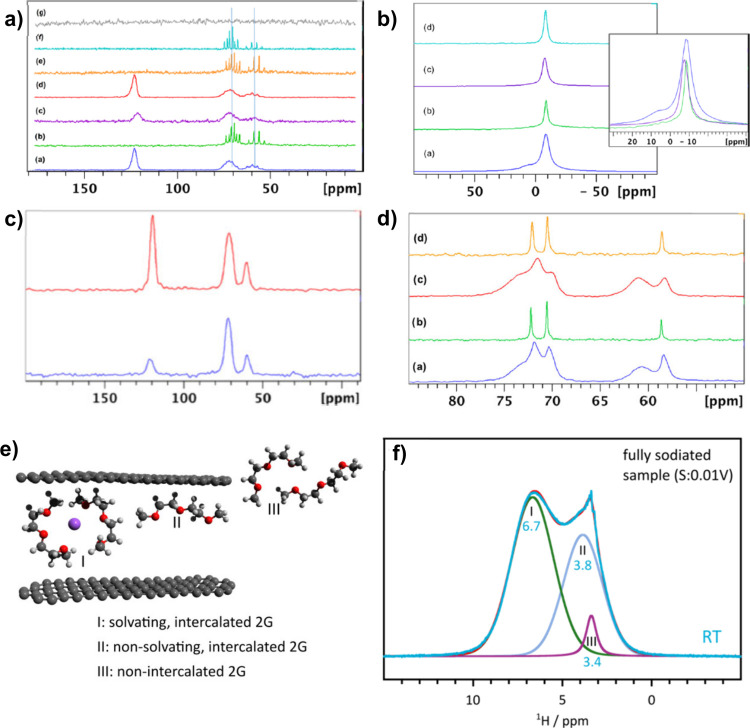
(a) ^13^C single-pulse MAS NMR spectra of graphite electrodes after three full cycles disassembled in the fully sodiated state (a, blue), fully desodiated state (b, green), partially sodiated state (charged to 0.3 V) (c, purple), fully lithiated state (d, red), and fully delithiated state (e, orange), along with a ^13^C single-pulse spectrum of the electrolyte NaClO_4_ in diglyme (f, cyan), provided for comparison and the spectrum of the uncycled graphite (g, gray). (b) ^23^Na single-pulse MAS NMR spectra in the fully sodiated state (a, blue), fully desodiated state (b, green), partially sodiated state (charged to 0.3 V) (c, purple), and 1 M NaClO_4_ in diglyme solution (d, cyan). (c) CP spectrum comparing the fully sodiated state (blue) and the fully lithiated state (red) at maximum contact time (9 ms). (d) ^1^H–^13^C INEPT spectra from samples with a fully sodiated state (blue), fully desodiated state (green), fully lithiated state (red), and fully delithiated state (orange). Reprinted with permission from ref (92). Copyright 2018 American Chemical Society. (e) Schematic representation of the different proton species and (f) ^1^H single pulse spectra from fully intercalated sodium ions in graphite using 1 M NaOTf in diglyme as electrolyte at room temperature (RT). Reproduced with permission from ref (93).

Leifer et al. have reported a pioneering work analyzing and detecting sodium and lithium co-intercalation with diglyme in graphite electrodes.^92^ First, by using ^13^C MAS NMR, they compared different charged and discharged electrodes (see Figure 9a). As can be seen, in the fully intercalated electrodes (blue and red lines), there is a shift appearing at 123 ppm. This stands in contrast to the deintercalated samples due to the interaction of sodium ions with the conjugated π-system upon co-intercalation, a similar effect to metal doping. In the case of the partially sodiated state (purple line), the shift is upfield by 2 ppm and broadened, which suggests decreased charge transfer and higher susceptibility. In addition, identical shifts of graphite intercalated compounds for the sodium and lithium systems are the result of similar susceptibilities. The peaks located at 59 and 70 ppm are caused by the presence of an electrolyte. The existence of a broad and downshifted signal for the intercalated compounds is due to diglyme molecules not occurring as a free liquid. Quantification to determine the ratio of carbon/diglyme present was also performed. Thus, the 70 ppm shift is due to the existence of aromatic carbon, while the 59 ppm shift is ascribed to aliphatic carbon. Escher et al. took this examination a step further and performed ^13^C MAS NMR measurements at various co-intercalation potentials.^93^ The findings indicate that at a low state of sodiation, there is a change in the solvation structure due to the presence of an additional peak at 135 ppm. This signal is suggested to originate from coordination between sodium with one diglyme molecule. When the sodiation continues, this peak downshifts to 125 ppm, indicating the coordination to two molecules. Not only lithium and sodium co-intercalation were studied by means of NMR. Park et al. used ^13^C NMR to demonstrate calcium co-intercalation with a DMAc-based electrolyte into graphite, where they compared the calciated electrode (i.e., Ca/DMAc into graphite to form *t*-GIC) with the pure liquid electrolyte.^63^ From these measurements, it can be concluded that NMR is a suitable tool for following the electrochemical co-intercalation reaction.

**Figure 10:**
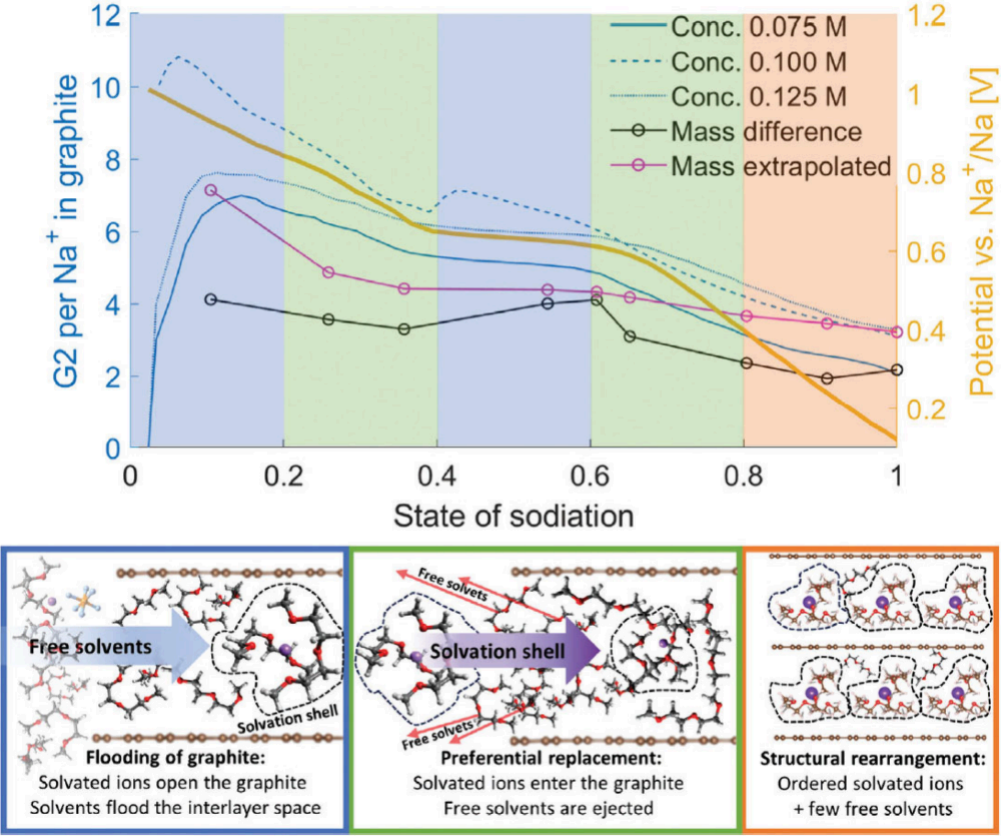
New model for solvent co-intercalation. (Top) The voltage profile and the ratio of 2G molecules per Na^+^ were measured with the mass experiments. (Bottom) Schematic illustration of flooding of the graphite, the preferential replacement of free solvents by solvated ions, and structural rearrangement into a more ordered structure. Reproduced with permission from ref (86).

Of particular interest for co-intercalation reactions could be also more specific NMR techniques such as pulsed field gradient NMR or electrophoretic NMR. These methods (while being difficult to apply for post-Li-ion chemistries) can provide information on the self-diffusion of ions and solvents as well as the transference numbers.^148,149^ These parameters can strongly vary in highly coordinated systems, e.g., when glymes are used a solvents.^150^ The use of these methods could therefore be effective in exploring the complex mechanism of co-intercalation reactions, especially when considering the situation of opposite flux of ions and solvents (see the model shown in Figure 10).

Other NMR measurements were conducted using MAS NMR. ^7^Li and also ^23^Na MAS NMR can provide valuable information, as reported by Leifer et al. and Escher et al.^92,93^ Thus, while measurements of the pristine electrolyte solution demonstrate a sharp and narrow peak (cyan signal; see Figure 9b), in the case of cycled electrolytes this peak needs to be divided into various contributions. In the case of deintercalated graphite, two peaks were obtained (one for the free electrolyte solution and another related to the sodium absorbed on the graphite surface, green signal). On the other hand, sodiated graphite measurements showed a reduced amount of free electrolyte solution, and a peak related to the intercalated electrolyte is the largest contribution of the spectrum (blue line). Lithium exhibited a similar interpretation of the results, although with three visible peaks.^93^^1^H–^13^C cross-polarization (CP) measurements were also used to prove co-intercalation.^92^ In this sense, the presence of diglyme methylene at 71 ppm and methyl at 60 ppm demonstrated that solvent molecules are inside graphite at the sodiated state (Figure 9c). Existence of the 123 ppm shift is further proof of co-intercalation, as it is the result of magnetic dipole–dipole interactions between electrolyte protons and graphite carbons. In addition, the higher intensity of lithiated versus sodiated carbon is the result of lower mobility of the lithium solvent inside graphite, with the solvated molecule being more tightly connected to the graphene sheets. Furthermore, ^1^H–^13^C insensitive nuclei enhanced by polarization transfer (INEPT) was explored to obtain information in the electrolyte/solvent region.^92^ In Figure 9d, interactions between diglyme and the outer surface of the electrode could be assigned to peaks at 58.4, 70.3, and 71.8 ppm, while the diglyme located in the interplanar graphite was detected through broad peaks at 60.8 and 72.4 ppm. Deintercalated samples exhibited only three sharp peaks: (i) 58.4 ppm, which is attributed to methyl carbon, and (ii) 70.2 and 71.6 ppm due to the methylene carbons from liquid diglyme. In addition to these NMR experiments, as can be observed in Figure 9e and f, ^1^H single-pulse NMR was used by Escher et al. to discern the state of different solvent molecules, specifically those solvated, not solvated but intercalated, and not intercalated.^93^ By reducing the temperature, the mobility of glymes co-intercalated into the graphite structure was demonstrated to be lower than that of the other two types of solvent molecules.

Finally, the metal-solvent interactions can also be analyzed by means of ^1^H–^7^Li CP NMR.^92^ In this case, a single line at 1.5 ppm was enough to confirm the interaction between lithium and diglyme. Like any other measurement technique, sample preparation is critical to obtaining reliable data. Thus, no solvent rinsing or drying was employed during the preparation of samples. Significant changes were observed when comparing samples with and without these preparation measures, highlighting that care must be taken to obtain correct information through sensitive measurement techniques.^41,93^

Prior to this Review, the discussion of the importance of solvent choice and its influence on the electrochemical performance was already presented. To elaborate on this topic, Escher et al. compared the co-intercalation mechanism in diglyme, triglyme, and pentaglyme by using ^23^Na NMR, ^1^H NMR, and ^1^H–^23^Na cross-polarization.^93^ Results showed that the mobility of triglyme molecules coordinated with sodium was reduced compared to that of the other glymes due to the increased interactions between solvent molecules and both graphene layers and sodium ions. Furthermore, it was concluded that diglyme-Na molecules exhibited the highest mobility among the co-intercalated species. This finding was corroborated by the much more favorable rate capability of the system, especially when compared with triglyme, where higher temperatures were needed to obtain similar capacity and rate capability.^8^

As has been established, NMR presents a valuable and convenient tool to obtain insights into the co-intercalation storage mechanism. Further investigations must be conducted to expand our knowledge of this complex analytical technique in relation to this topic. Finally, the development and implementation of an in situ setup to monitor changes within the electrochemical system during cycling can be considered a top priority in the NMR field.

### 4.8 Mass Measurements

Mass or weight measurements represent ex situ measurement techniques where the mass of cycled electrode is monitored versus the pristine electrode material. Since the amount of cations inserted can be calculated from the maximum specific capacity (for a system with an excess of electrolyte), a simple comparison to the mass of the electrode before and after intercalation will serve to determine the ratio between solvent electrolyte and cation. This straightforward approach has been used in graphite electrodes with Li,^10,91,151^ Na,^152^ K,^90^ and Ca,^153,154^ TiS_2_ electrodes with Na,^72^ and conjugated polymer with Na.^79^ Even though the simplicity of this method makes it appealing for use, the pretreatment of the sample can be rather complicated. Once the cell is disassembled, the question arises around what the electrode weight entails. The simple answer is that the mass of the cycle electrode is composed of only the pristine electrode plus the intercalated species. However, the remaining electrolyte solution can be still on the surface, and this can lead to significant calculation errors. Several authors have proposed a drying procedure in order to remove the excess electrolyte from the electrode.^72,152^ However, in our previous work, we demonstrate that this process will evaporate not only the solvent electrolyte but also the solvent co-intercalated in the interior structure of the electrode.^86^ Thus, the remaining excess mass will consist mainly of the salt from the electrolyte with no solvent co-intercalated molecules in the structure, giving way to wrong interpretation of data.^86^ In this regard, our approach considers both the mass of the intercalated and the deintercalated samples, hence accounting for the mass of the intercalant and the mass of the excess electrolyte, the latter being similar for both samples (since the open structure area is analogous). In addition, two methods were developed in order to obtain the most accurate mass of co-intercalated solvent. The first method (mass difference method) compares the masses immediately after the cell has been opened. The second one (mass extrapolation method), on the contrary, analyzes the time-dependent drying of the electrode considering the different nature of the solvent electrolyte molecules. This work demonstrated that the storage mechanism is far more complex than previously presented, as during the electrochemical reaction the ratio between the solvent electrolyte and cations changes and is not a constant value, as commonly thought.^152^ A new model was proposed in which the graphite electrode is flooded by free glymes once the first solvated ions intercalate into the structure at the beginning of the electrochemical process. As the sodiation continues, the free solvents are replaced with solvated ions until the main plateau. Once the graphite is fully sodiated, a structural rearrangement into the final structure occurs, consisting of mostly solvated ions with a few free solvent molecules. The variation of the glymes per cation intercalated in the graphite structure with the state of sodiation is displayed in Figure 10 along with the proposed model.

To conclude, mass measurements represent a very useful technique to detect changes that can correlate to the stoichiometry of the reaction. Since the co-intercalation leads to the insertion of both the cation and the solvent molecules, a significant mass increase is expected in a co-intercalated system compared to a conventional intercalated one, making it possible to detect the co-intercalation mechanism. However, it is important to note that this is an ex situ technique, which can be prone to errors from handling, miscalculations, or misinterpretation of the results. To get more insight on the analysis technique as well as the calculation of the different ratios and the possible errors with the results, the reader is referred to the work by Åvall et al.^86^

### 4.9 Additional Techniques

Up to this point, the most important measurement methods to investigate the co-intercalation mechanism and its differences from other electrochemical processes were discussed. Nevertheless, those can be complemented with additional techniques in order to gain deeper insight into the intricacies of the co-intercalation process. In this subsection, a brief assessment of these methods and their importance will be provided.

#### 4.9.1 Fourier Transform Infrared Spectroscopy (FTIR)

**Figure 11:**
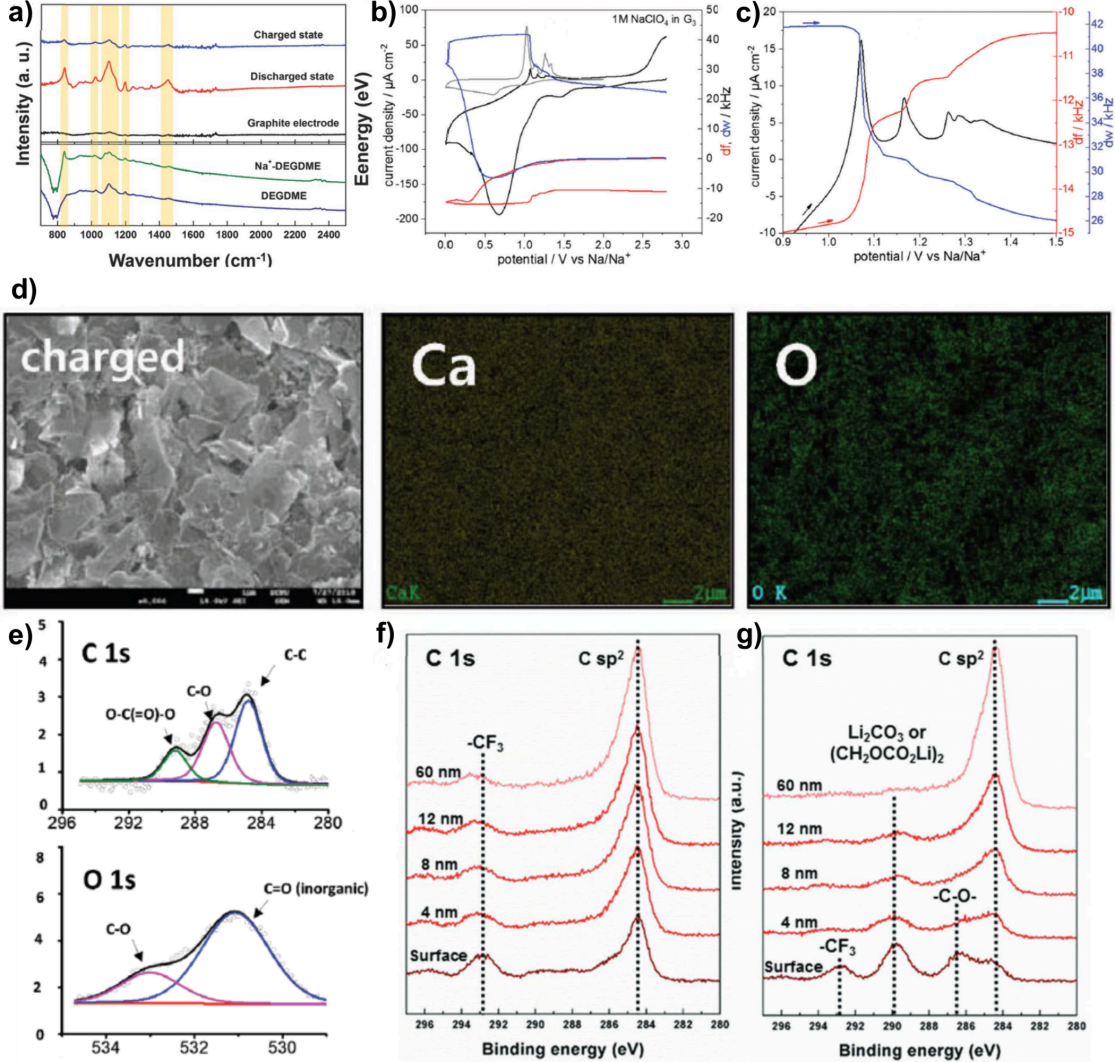
(a) FTIR analysis of different states of sodiation in graphite electrode and 1 M NaPF_6_ in diglyme. Reproduced with permission from ref (130). (b) In operando EQCM data for graphite coated on Au-quartz and (c) magnified desodiation peaks in 1 M NaClO_4_ triglyme. Reproduced with permission from ref (124). (d) Field emission scanning electron microscopy and EDX images in a graphite electrode and 1 M Ca(TFSI)_2_ in tetraglyme. Reproduced with permission from ref (64). (e) XPS characteristics of the ether-derived SEI in graphite electrode with 1 M NaPF_6_ in diglyme. Reproduced with permission from ref (109). XPS analyses characterizing the edge of graphite cycled with (f) 1 M LiTFSI in diglyme and (g) 1 M LiTFSI in EC/DMC (1:1, v/v). Reproduced with permission from ref (58).

Infrared spectroscopy allows one to identify functional groups in a given electrode/electrolyte. To achieve this, the spectra of both intercalated and deintercalated electrodes can be compared to the characteristic peaks of the pristine electrolyte mixture. The existence of solvated molecule features on the spectra obtained for the solid electrode can be considered as a sign of co-intercalation behavior. Kim et al. reported the presence of the vibration peaks of the solvated sodium molecules in the sodiated graphite electrode, which was suggested to be co-intercalation into graphite (see Figure 11a).^130^ In a similar way, Zhu et al.^57^ and Kim et al. (for Mg co-intercalation)^25^ took advantage of this technique to elaborate on the reaction mechanism. Once again, the prior discussion on drawbacks and advantages of ex situ experiments can be included here.

#### 4.9.2 Operando EQCM

Generally, EQCM (electrochemical quartz crystal microbalance) is considered to be a highly sensitive detection method for any changes in weight up to a range close to nanograms. While utilizing the inverse piezoelectric effect, it is possible to correlate the sample mass to the resonant frequency. Therefore, mass changes due to intercalation or co-intercalation could be detected to gain further insight into the mechanistic properties of the intercalation process or the surface layer evolution. Application of EQCM requires an elaborate setup and specific conditions, which must be met to obtain interpretable mass change data. Limitations of the Sauerbrey model to interpret EQCM data are strict and only fulfilled for acoustically thin, homogeneous, and rigid films, which is not the case for standard battery electrodes.^155^ The first practical hurdle is that casting electrode particles on a quartz crystal leads to a porous film. In addition, the co-intercalation reaction is a process that causes variations in electrode thickness, porosity, and surface area, all of which present challenges when applying the Sauerbrey model. Application of other models that take morphological parameters into consideration as suggested by Daikhin et al. can only provide qualitative assessments^156,157^

To the best of our knowledge, only one publication so far has employed EQCM for an investigation of co-intercalation in respect to sodium-ether complexes in graphite (see Figure 11b and c).^124^ Though the results could not be interpreted quantitatively, the authors found that the EQCM signals showed a correlation between the appearance of anodic peaks and a frequency increase proportional to the peak of charge. This suggested that desodiation in a triglyme system could be a suitable example of applying EQCM in the battery field and should be investigated with particles of homogeneous size, equally distributed, and hemispherical. In the last years there has been no progress on this topic, though the successful application of EQCM could add a valuable contribution to the investigation of the observed asymmetric diffusion properties and to the discussion of coordination properties of different glyme-based electrolytes.^22^ Finally, this method presents a promising approach to complement the results obtained with other techniques such as mass measurements, impedance, NMR, XRD, or ECD to shed more light on the co-intercalation mechanism.

#### 4.9.3 Energy-Dispersive X-ray Spectroscopy (EDX)

Although EDX is usually coupled with other microscopy instruments (TEM, SEM), it is a nondestructive X-ray technique used to determine the elemental composition of the electrode surface. By using an energy dispersive detector, it is possible to analyze the X-ray signal produced by the SEM (or TEM) electron beam.^158^ Thus, with this technique, cation contents can be revealed at both the intercalated or deintercalated stages.^57,64^ In the work of Prabakar et al., EDX was used to determine the atomic ratios of O/Ca in the intercalated sample to prove the co-intercalation of tetraglyme with calcium (see Figure 11d).^64^ Similarly, Kim et al. calculated the ratio of O/Ca to be 3 when using diglyme as the solvent electrolyte, which led to a ratio of one solvent molecule per sodium ion.^83^ Nevertheless, prior considerations of ex situ techniques should be taken into account. In addition, the accuracy of this analysis depends greatly on the homogeneity and surface quality of the sample. Therefore, special attention is required when discussing the ratios of elements to avoid drawing any premature conclusions. When using EDX, it is highly recommended to confirm the sample composition with other complementary techniques (elemental analysis, XPS, etc)

#### 4.9.4 X-ray Photoelectron Spectroscopy (XPS)

Finally, XPS represents another surface analytical tool that provides the possibility of examining the electrode surface chemistry. With this technique, not only the chemical composition but also the chemical nature can be analyzed. Although it is more widely employed for SEI investigation, some groups have reported its use to be similar to that of EDX.^64,130^ However, XPS is a very surface-sensitive technique (1–10 nm),^159,160^ which raises the question of whether this can be an appropriate method to reveal solvent co-intercalation. Similar to TEM or SEM, the preparation of the samples is quite challenging as the samples are easily modified.^160^ For instance, electrode rinsing and drying have been considered a necessary step before introducing the sample into the instrument in order to avoid misinterpretation of the results due to remaining electrolyte on the surface. However, rinsing with certain solvents might result in altering the electrode chemistry or even washing off a thin surface layer formed, thus resulting in unreliable measurements. As a result, data analysis from this technique must be handled with certain caution. It is our belief that this instrument might not be among the most appropriate methods to investigate solvent co-intercalation in batteries.

As already mentioned, this technique has been widely used for the determination and characterization of SEI. In the case of the co-intercalation reaction, the absence or existence of a relatively thin surface layer makes its detection and characterization quite challenging. In literature, reports of both the presence and characterization (composed by organic and inorganic compounds)^109,161^ as well as the nonexistence of SEI (in comparison with other electrolytes)^58^ through XPS measurements have been published (see Figure 11e–g). It again seems necessary to emphasize the importance of proper sample preparation prior to conducting sensitive measurement techniques.^160^ Additionally, the existence of other carbon additives that can form an SEI as well as the use of different and/or unstable conductive salts might be the reason for certain contradictory results.^66,109,161^

## 5 Theoretical Investigations

Theoretical studies serve as an important complement to the experimental studies and can be used to reveal the finer details of the reaction mechanism. Several papers have tried to pinpoint key metrics to determine if a solvent co-intercalation reaction is at all possible between a given electrolyte and electrode material. Most of these studies have focused on the electrolyte properties, especially the solvation/desolvation free energies or the electrolytes stability toward reduction.^41,42,61,86,126,133,162−165^ A few have studied properties of the electrode material, such as the interlayer binding energy.^86,89^ As of yet, however, there is neither a validated systematic approach to theoretical investigations into solvent co-intercalation phenomenon nor a clear consensus on what the key determining factors that enable the reaction are.

### 5.1 Electrolyte Investigations

**Figure 12:**
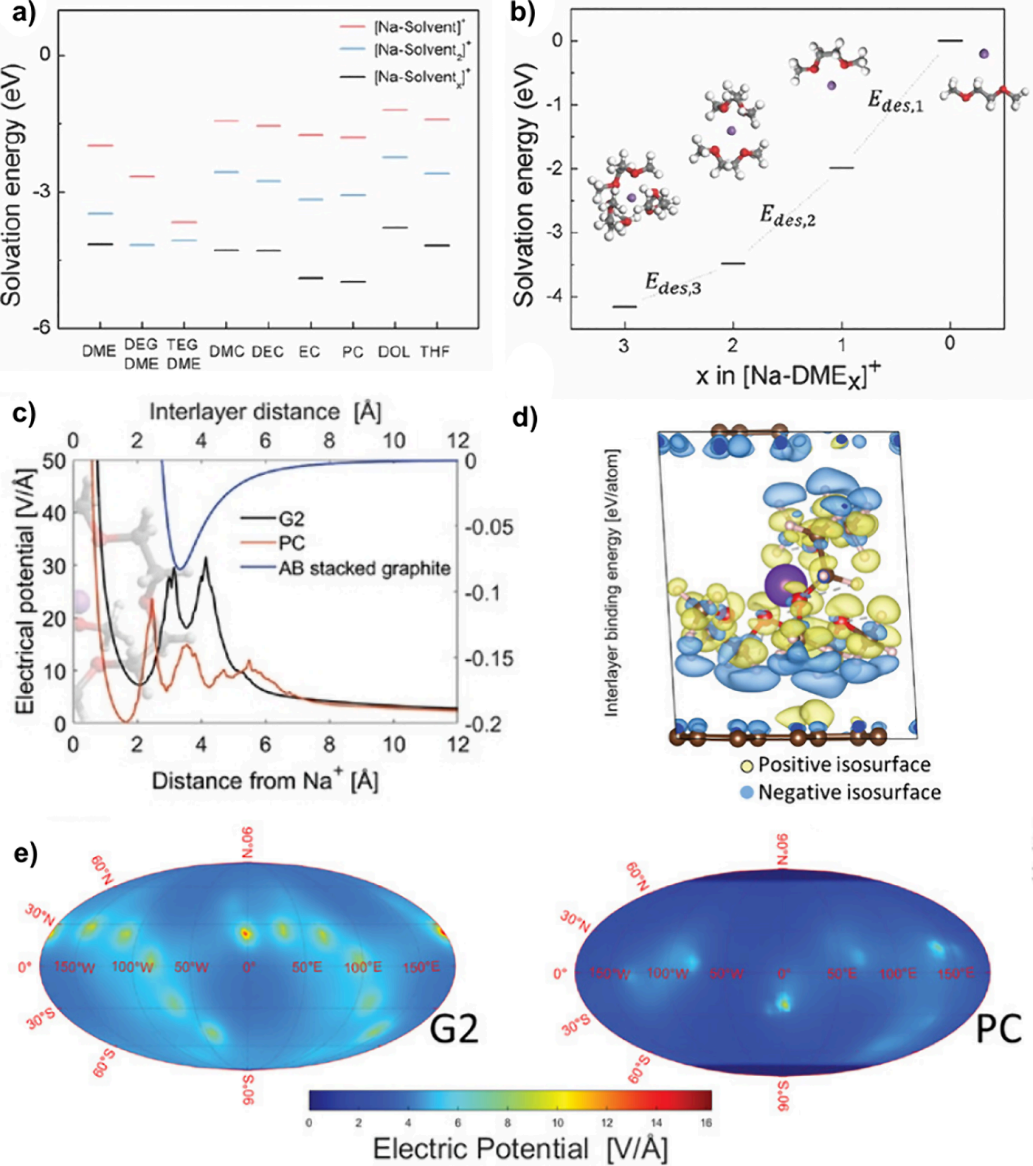
(a) Solvation energy of Na-solvent complexes and (b) energy diagram of the [Na-DME_*x*_]^+^ desolvation process. Reproduced with permission from ref (126). (c) The electrical field strength at a distance from Na^+^, averaged over the entire sphere around diglyme (G2) (solid black) and PC (orange), as well as the interlayer binding energy of AB stacked graphite (blue). (d) The electron density difference of a stage I t-GICs with G2, with blue (decrease in electron density, more positive) and yellow (increase in electron density, more negative) 0.3 e Å^–1^ isosurfaces drawn. (e) The Mollweide projection of the electric field averaged along solid spherical angles of G2 (left) and PC (right); note the atoms of the solvent molecules can be identified by the bright spots in the figures. Reproduced with permission from ref (86).

In an early work on reversible electrochemical co-intercalation, Yoon et al. highlighted the importance of the stability of the solvation shell, as well as the reductive stability of the solvent, for reversible co-intercalation to occur (Figure 12a and b).^126^ As the process of intercalating a full/partial solvation shell requires a large and energetically costly expansion of the host structure, the solvation shell must be very stable such that the cost of removing the solvation shell rivals the cost of expanding the host structure. Thus, solvents that tend to coordinate strongly to ions are prime candidates for solvent co-intercalation. Consequently, several papers on solvent co-intercalation have utilized density functional theory (DFT) to investigate solvation/desolvation free energies of ethers and Na^+^,^41,42,86,126,162^ as well as for Li^+^^162,163^ and K^+^.^61,133,163,164^ Interestingly, although 1,2-diethoxyethane (DEE) and 1,2-dimethoxyethane (DME, also known as monoglyme, 1G) only differ in the methyl end group of DEE, DEE does not co-intercalate into graphite with K^+^, although DME does, which the authors attribute to the stronger solvation of DME.^163^ Similarly, PC and ethylene carbonate (EC) only structurally differ by a methyl group, but the small difference in solvation energy might be the root cause to PC more effectively co-intercalating into graphite, although exfoliating and delaminating the structure in the process. A few papers also emphasize the importance of the solvation shell being not only stable but also small,^42,82,83^ the argument being that the smaller the solvation shell, the less the structure needs to expand to accommodate the solvated ion; hence, the energy of expanding the structure is reduced, making co-intercalation more favorable.^42^ In addition, a smaller solvation shell brings the host structure material closer to the solvated ion, leading to a stronger interaction between the solvation shell and host structure, which further promotes the reaction (Figure 12c–e).^86^

Due to the solvents being in constant contact with the interior of the electrode active material, the stability of the solvent toward reduction was also identified as a key descriptor for reversible co-intercalation.^126^ If the potential of the solvent co-intercalation reaction is higher than the reductive stability of the solvent, co-intercalation can occur before any SEI-forming reactions. Although there are schemes to calculate the reduction potential of solvents, carried out, for instance, for 2G,^166^ the lowest unoccupied molecular orbital (LUMO) is very often used as a proxy for reductive stability. However, it should be noted that the use of the highest occupied molecular orbital (HOMO) and LUMO instead of the oxidation and reduction potential of the electrolyte is problematic as they are fundamentally different from the oxidative/reductive potential of the solvent^167^ and show poor correlations with it.^168^ Instead, one study carried out ab initio molecular dynamics (AIMD) to study the electrolytes interaction with the edge and basal plane of graphite, showing how an electrolyte composed 1 M LiBF_4_ in 1G reacts with the edge plane but not with the basal plane of graphite, passivating the catalytic edge sites.^165^ However, due to the low effort of computing the HOMO/LUMO in a standard computational procedure, it is often the analysis carried out. The LUMO level is then compared with the Fermi energy of the electrode active material to assess their compatibility. However, the absolute value of the LUMO level, as well as the Fermi energy, is strongly dependent on the level of theory it is computed at, as can be observed in Table 1. Considering that often the Fermi energy is computed with periodic DFT while LUMO levels of solvents or solvated ions are in general computed using nonperiodic DFT, it seems unclear if the LUMO can be used as a useful descriptor in the hunt for electrolytes showing solvent co-intercalation.

**Table 1:** LUMO Levels of THF and 2G Calculated with the Two Popular Functionals B3LYP and M062X with and Without the SMD Implicit Solvent Model^a^

solvent	LUMO_B3LYP_ [eV]	LUMO_M062X_ [eV]	LUMO_B3LYP_^SMD^ [eV]	LUMO_M062X_^SMD^ [eV]
THF	–1.13	–1.42	–5.30	–5.70
2G	–0.75	–0.92	–4.04	–4.34

a Calculated on the 6-311++G(d,p) basis.

However, in the end, the solvent must be reductively stable in the environment deep inside the charged graphite gallery, as otherwise the system would have either delaminated, become passivated, or shown great irreversible capacities; neither is observed.

The structure and dynamics of the solvation shell are of particular importance for solvent co-intercalation. In the case of glyme-based electrolytes the solvation shell has been studied theoretically in several different ways, such as molecular dynamics,^41,169−183^ along with several spectroscopy studies combined with DFT often originating from the polymer or ionic liquid community or from studies related to metal-air batteries.^61,184−188^ Generally, these show that the solvation shells composed of glymes are extremely stable, and solvent molecules are present in the solvation shell even at high salt concentrations. However, generally, it is hard to quantitatively investigate the content of the solvation shell using IR/Raman spectroscopy due to the great number of conformations with unique IR/Raman signatures.^61,185−190^

### 5.2 Electrode Investigations

Several studies have looked at solvation shells inside graphite, including those for Li^+,^^162,164,191−193^ Na^+,^^82−84,86,162,191,194^ K^+^,^61^ Mg^2+^,^25^ and Ca^2+^,^63^ and a few studies have been carried out on solvated ions in TiS_2_ and NaTiS_2_.^69,89^ Of these, the majority study either fully or partially solvated ions, while a few make a comparison of these. In addition, a few molecular dynamics studies have been carried out where the number of solvent molecules in the host material has not been determined from the start.^69,194,195^

Three in-depth DFT studies stand out for having investigated several possible structures with solvated ions in graphite,^83,162,191^ including a comparison between Na^+^ and Li^+^,^162,191^ and different glymes.^83^ Kim et al. studied partially solvated Na^+^, i.e., solvated by a singly glyme molecule, and compared stacking severally partial solvation shells inside the graphite interlayer, finding that double stacking with the Na^+^ between the graphite layer and the glyme molecule produces an interlayer distance in good agreement with XRD measurements for 1G, 2G, and 4G.^83^ Moreover, they found that having Na^+^ aligned along the *c*-axis produces the most stable structures and hence these should form first. If the Na^+^ are not aligned, less stable structures are formed that also give rise to several low intensity peaks in the 11–22° region in an XRD spectrum, which the authors correlate with the in-plane superstructure identified in the same paper forming at low potentials vs Na^+^/Na.^83^ Following this, Jung et al. used AIMD to generate starting structures used for geometry optimization and concluded that both [Li:2G]C_16_ and [Na:2G]C_16_ are stable compounds.^191^ Moreover, both nudge elastic band calculations as well as AIMD simulations show that the sodium complex has a diffusion coefficient 5 orders of magnitude greater than the complex, in agreement with the experimentally observed kinetic differences between lithium and sodium. The same differences in diffusivity were not observed when the ions were coordinated to two 2G molecules and hence the coordination to a single 2G molecule was deemed more probable.^191^ Jung et al. also showed that it is more difficult to exfoliate the graphite once the solvated ions are inside and that there is considerable interactions between the solvent molecules and the graphene layers, which was also noted in other independent studies.^83,86,162^ In contrast, when Yu et al. investigated differences between Li^+^ and Na^+^ in *t*-GIC, with several possible alkali metal to carbon ratios, as well as both fully and partially solvated ions, it was found that [Na:(2G)_2_]C_21_ (∼100 mAh g^–1^) is the most energetically stable compound and favored over structures with partially solvated ions.^162^ Moreover, they investigated the performance of several common functionals and showed the importance of including van der Waals corrections.

There is also an MD study that directly simulated solvent co-intercalation into hard carbon and Na^+^ solvated by DMSO in graphite; however, solvent co-intercalation has not experimentally been shown to occur into hard carbon, and neither has electrochemical co-intercalation of Na^+^ into graphite in a DMSO-based electrolyte.^195,196^

**Figure 13:**
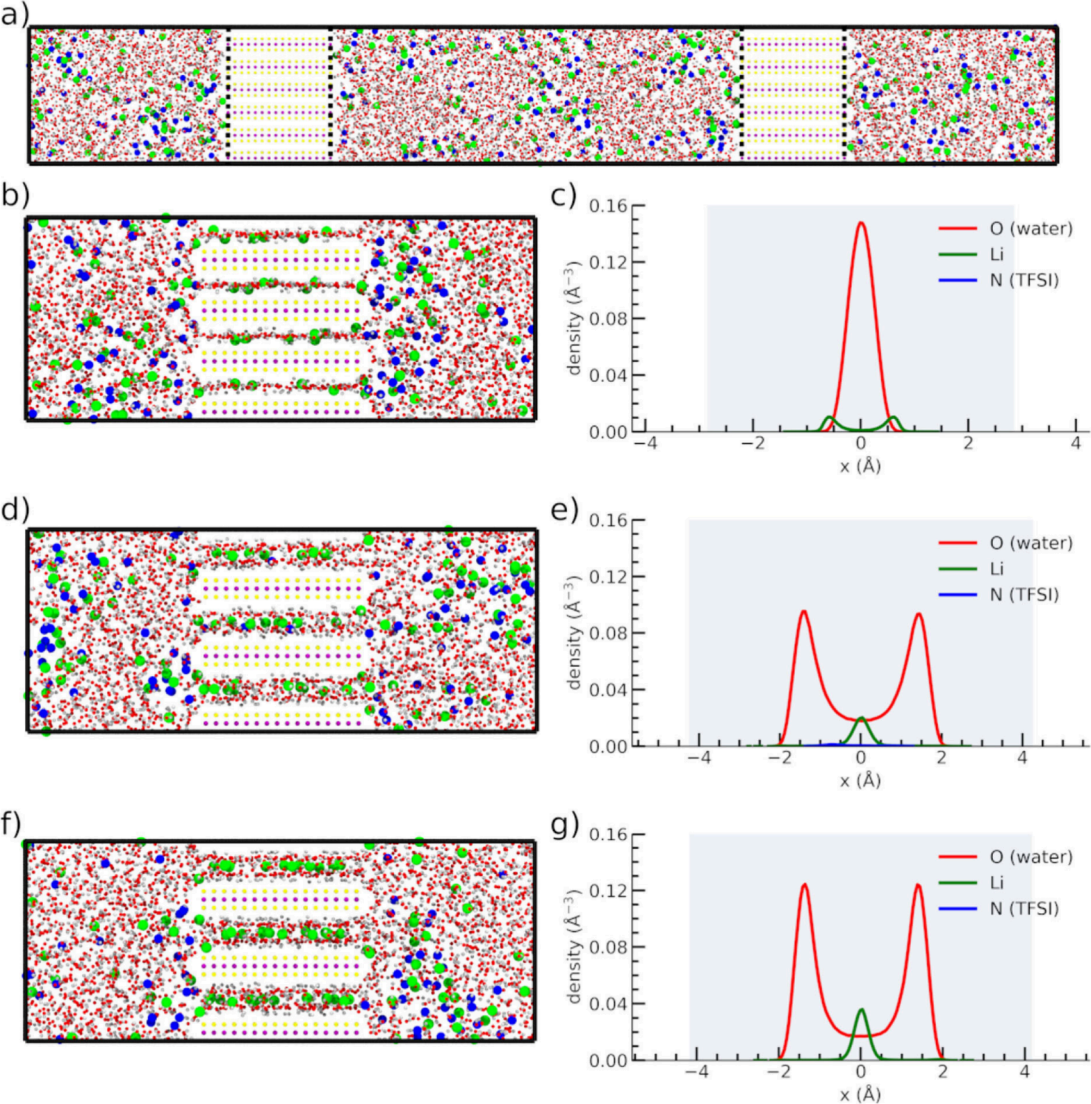
Snapshot of the MD simulation cell of TiS_2_ with an aqueous electrolyte (a) for an interlayer distance of 5.7 Å at the OCV, with oxygen (red), hydrogen (white), Ti (purple), S (yellow), and Li (green). For TFSI, only the N atom (blue) is shown. MD snapshots and atomic density profiles of the working electrode plotted at (b, c) 8.52 Å and −0.13 V vs SHE, (d, e) 11.34 Å and −0.19 V vs SHE, and (f, g) 11.18 Å and −0.65 V vs SHE, corresponding to the m, d, and d′ phases, respectively. The density profiles are computed along the direction of the interlayer spacing and averaged over all the interlayers of the electrode. The pore size, i.e., interlayer distance, is highlighted by the light blue background in (c), (e), and (g). The origin is set at the position of the Ti plane. Reproduced with permission from ref (69).

None of the above studies included any free solvents inside the graphite structure, for which there is ample experimental evidence for. Most strikingly, every NMR study so far conducted has shown that a considerable amount of free solvents is present along with solvated ions inside the graphite structure.^92,93,147^ Notably, two classical molecular dynamics studies, one using ionic liquids and graphite and the other using an aqueous electrolyte and TiS_2_, Figure 13, show that when solvent co-intercalation occurs not only solvated ions but the other components of the electrolyte enter the solid structure (although anions do not enter, or enter in very small amounts).^69,194^ Moreover, there is also a lack of studies that properly resolve stacked graphite structures with a correct stoichiometry, although to carry out such calculations very large structures would have to be used at a heavy computational cost.

To conclude, the majority of atomistic simulations make use of DFT, where it is well-known that the result of such calculations depends heavily on the initial configuration guessed by the researcher; this starting configuration is based on simply the experience of the scientist. In the case of solvent co-intercalation, this basic intuition is often missing, including even basic facts such as the number of solvents per ion inside the active material or how the solvents and ions are orientated relative to the host structure. Therefore, it is not surprising that a great deal of possible structures are presented in the literature. Moreover, the majority of studies have focused on either electrolyte properties in isolation or at a single solvation shell inside a host material. However, it is clear that the solvent co-intercalation phenomenon is determined by the interaction between the electrolyte and the electrode active material; therefore, the interface where these two phases meet is of outmost importance, and neither the electrolyte nor the electrode active material should be studied in isolation. It is therefore the authors’ opinion that computational studies where the electrode and electrolyte interface are both resolved are crucial to advance the field, yet these are rare (Figure 13).^69,165,194,195^ However, they are truly needed in order to resolve the internal structures of a charged host structure filled with solvated ions and possibly free solvents. Once the research field has identified the probable internal structure of an active material filled with solvated ions, as well as free solvents, a more thorough deconvolution of the redox potential can be carried out. Hopefully, this would allow the research field to identify key descriptors for predicting when a solvent co-intercalation reaction is possible and hence direct the community to what combination of solvents and host structures allow these reactions.

## 6 Key Characteristics of Electrochemical Co-Intercalation Reactions

### 6.1 Advantages

The unique properties of solvent co-intercalation reactions result from the specific and complex interactions between the type of host structure, the type of intercalated ion, and the type of solvents. Due to the wealth of possible combinations, it is difficult to define general advantages for this type of reaction; however, some benefits related to that are (a) the redox potential can be tuned by careful choice of the type of solvent and salt, including their ratios, and (b) the diffusion of solvated ions can be very fast, which, combined with (c) the absence (or presence of a very thin and low resistive) SEI, may enable the development of high power devices. The special interaction between the host material and the inserted ions and solvents can also enable a very good cycle stability of over several thousand cycles despite the comparatively large expansion of the electrode. While these advantages are especially apparent for co-intercalation of Na^+^ and glymes in graphite, the situation is not as clear for co-intercalation in cathode materials such as layered sulfides.

#### 6.1.1 (Initial) Coulomb Efficiency

By using ether-based electrolytes with graphite, the co-intercalation process exhibits a similar or even higher Coulomb efficiency compared to common intercalation processes with carbonate-based electrolytes. Generally, the Coulomb efficiency describes the ratio between the charging and discharging capacity, with the initial Coulomb efficiency (ICE) referring to the Coulomb efficiency of the first charge/discharge cycle. Values close to 100% indicate the high reversibility of the reaction, while values deviating from 100% indicate capacity losses and/or side reactions. ICE values for intercalation anodes are typically below 100%, since in the first cycle SEI formation takes place (see further below for more details).

For the co-intercalation reaction of Na^+^, diglyme and graphite, Jache et al. reported in 2014 an irreversible capacity of 10–15 mAh g^–1^ with specific capacities close to 110 mAh g^–1^.^19^ The high ICE in the range of 90% combined with an overall Coulomb efficiency of 99.87% over 1000 cycles indicated a very high reversibility of the reaction. Values for ICE can be further increased by heat treating the graphite to remove the surface groups. On the contrary, the addition of 20 wt % Super-P as a conductive carbon to the graphite electrode decreases the ICE down to 64.9%, which can be ascribed to decomposition reactions on the conductive carbon during the first cycle.^197^ Further, Liu et al. found in 2019 a connection between the NaPF_6_ concentration of the diglyme- based electrolyte and the ICE.^108^ The lowest ICE of 68% is reported for 2.5 M NaPF_6_, along with that of 80% for 0.1 M NaPF_6_, indicating a NaF-rich surface layer causes the side reactions and an interphase of higher resistance. Therefore, a non-negligible amount of irreversible capacity loss in the first cycle can be attributed to an inorganic NaF-rich SEI evolving, that also has a profound impact on the impedance behavior of the cell.

Considering TiS_2_, ICE values for co-intercalation are typically lower compared to intercalation. For example, the ICE for co-intercalation of diglyme was inferior (65%) compared to common intercalation of THF (91%) and EC:DEC (88%).^42^ In this case, the low ICE was due to not only the expansion on the structure but also by side reactions due to the carbon additive.^42^ Nevertheless, the Coulomb efficiency approached 100% over prolonged cycling.

#### 6.1.2 Solid Electrolyte Interphase (SEI)

The concept of SEI formation is very well established in the field of Li-ion batteries.^198^ While being highly resistive to electrons, the SEI ensures the transport of ions to prevent the decomposition of the electrolyte and enables a continuous electrochemical charge and discharge of the battery. SEI formation takes place as a result of electrolyte decomposition once the electrode reaches too reductive potentials, i.e., typically for values below 1.0 V vs the alkali metal electrode. Some of the decomposition products are solid, forming a continuous surface film on the electrode. This film can lead to a passivation of the electrode, preventing any further electrolyte reduction and co-intercalation of solvents but still enabling ion conduction, hence the name solid electrolyte interphase.^199^ The decomposition reactions during SEI formation are complex and include, next to solid products forming the SEI, the formation of gaseous compounds.

A key question related to co-intercalation reactions on anodes such as graphite is whether SEI formation takes place. As mentioned, the concept and the observed low polarization of the electrode indicate an SEI-free (or nearly SEI-free) interface, which is against the common view that SEI formation is needed to prevent continuous electrolyte decomposition. Moreover, the large volume change of co-intercalation reactions should lead to rupture of the SEI and therefore again to continuous electrolyte decomposition during cycling, leading to low values for the Coulomb efficiency. The opposite, however, is found, and graphite shows excellent cycle life and efficiency over thousands of cycles for co-intercalation reactions. Gas analysis during cycling did also not show any excessive decomposition reactions during cycling, indicating a stable interface after an initial cycle were some side reactions take place.^66^

Overall, reports on SEI formation on co-intercalation graphite electrodes are not conclusive yet. While SEI formation has been reported by some groups,^108,109,124,130,161^ other studies did not find any indication for SEI formation.^82,139,200^ Note that surface film formation may also be due to side reactions caused by corrosion of the sodium counter electrode.^66^

From our own current perspective, there is either no SEI or an extremely thin one that is very flexible to allow the high-volume expansion of the electrode and very conductive and would need to have very different properties compared to a conventional SEI. Any crack on the SEI during the intercalation/deintercalation might lead to a further reduction of the electrolyte. However, as mentioned before, using online electrochemical mass spectroscopy, gas release was only found in the first cycle.^66^ An important argument for the SEI-free interface is the very high rate capability of the reaction and the negligible polarization of the electrode, especially at potentials close to 0 V vs Na^+^/Na. The side reactions occurring during the first cycle may largely lead to soluble or gaseous products, thus leaving a relatively clean interface rather than a SEI. One also has to realize that the existence of an extremely thin SEI is very difficult to prove experimentally. Surface studies are nevertheless needed and comparative studies by Wang et al. on graphite electrodes provide at least indication that an ether-derived SEI is much smoother and flexible compared to the carbonate-derived one.^109,130^ At the same time, one has to keep in mind the general challenges of preparing samples for (ex situ) surface-sensitive methods such as XPS (see section 4), the presence of conductive carbon additives,^109^ as well as the surface film formation derived from corrosion side reactions of the counter electrode.^66^ The lower irreversible capacity of the co-intercalation reaction compared to conventional intercalation can be indicative of evident differences in the amount of decomposition products formed in the first cycle, which could be the reason for the thinner surface layer.^82,109^ The degree of SEI formation can also be followed by electrochemical impedance spectroscopy, which also gave an indication of a lower interfacial resistance for glyme electrolytes (co-intercalation) as compared to carbonate electrolytes.^109^ This low resistance contributes further to the very good high-rate performance and reversibility over several thousand cycles.^20,57,82^

On the following subsections, the origin of SEI formation in relation to the co-intercalation reaction with conventional electrolytes will be discussed and revisited.

##### 6.1.2.1 SEI Formation in Conventional Electrolytes: The 3D Model Revisited

**Figure 14:**
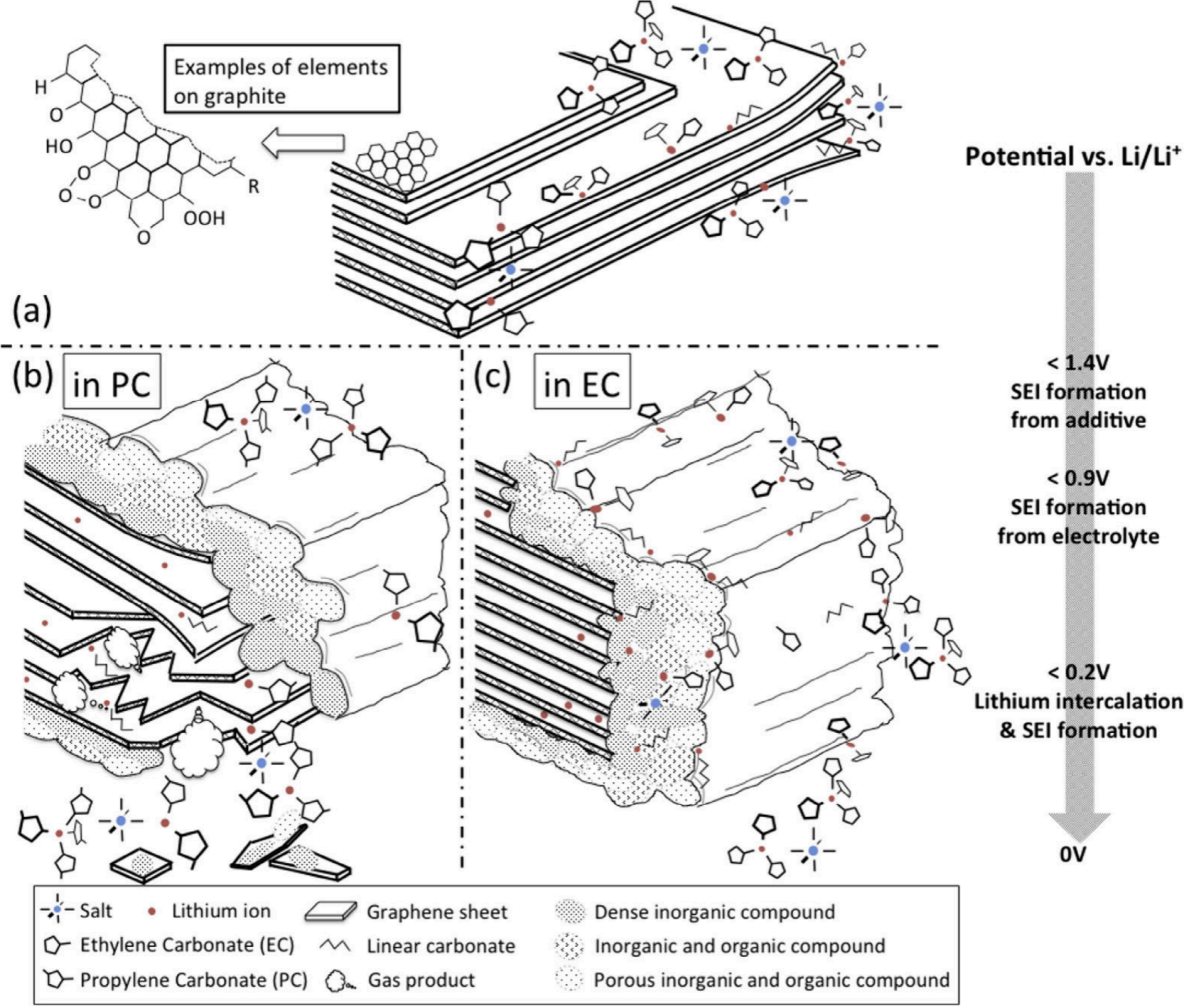
Schematic of the anode SEI formation process showing (a) graphene layers surrounded by electrolyte salts and solvents above 1.4 V, (b) propylene-carbonate (PC) intercalation with lithium ions into graphene layers and resulting exfoliations below 0.9 V, and (c) stable SEI formation in ethylene-carbonate (EC)-based electrolyte below 0.9 V. Reproduced with permission from ref (199).

Before EC became the predominant solvent used in LIBs, electrolytes containing PC were standard, and it was under this era that SEI formation on graphite started to be studied. In an early work by Arakawa et al. the well-known propane gas evolution that occurs when graphite is cycled in a PC-containing electrolyte was found to be inconsistent with the known PC decompositions pathways.^15^ To resolve this inconsistency, Arakawa and co-workers suggested the formation of an intermediary, a GIC where the lithium is intercalated along with its solvation shell, and showed how this explains the observed gas evolution rate. The suggested intermediary later received criticism in the seminal work of Jeff Dahn.^201^ But, to the best of our knowledge, this is the first time solvent co-intercalation was mentioned as a step in the electrolyte decomposition process. Soon, Besenhard et al. showed using dilatometry that there is indeed solvent co-intercalation occurring in early stages of SEI formation in both PC-based and EC:DME-based electrolytes.^13,16^ These studies laid the foundation to what is now known as the Besenhard–Winter 3D model of SEI formation on graphite (Figure 14), whereby the process is initiated by solvent co-intercalation occurring at the surface edge sites of graphite, creating a highly reductive environment in which, depending on the solvent, graphite exfoliation or successful stable SEI formation occurs.^202^

At that point, however, the case for co-intercalation occurring as a precursor to SEI formation came mainly from studies using electrolytes that are known to cause solvent to co-intercalate, i.e., they either contain glymes or PC. However, investigations from Petr Novák′s group showed that co-intercalation indeed also occurs in EC-based electrolytes during the first stages of SEI formation, strengthening the case that this generally occur during SEI formation on graphite.^203,204^ In particular, heat-treating the graphite in order to remove surface functionalities and thus reduce the reactivity with EC would cause solvent co-intercalation and exfoliation in an EC electrolyte, showing that the electrochemical stability of the solvation shell is crucial for reversible solvent co-intercalation. Nevertheless, the evidence for the 3D model is much scarcer in studies with greater reversibility (in systems with reversible intercalation, evidence of solvent co-intercalation at the graphitic surface during the start of the formation cycle would naturally be hard to come by, as successful SEI formation should block solvent co-intercalation) and has received criticism. Most strikingly, there is a lack of XRD evidence for *t*-GIC formation preceding SEI formation,^205−208^ although it was detected by XRD by Wagner et al.^16^ However, it has been argued that this is not surprising, as the subsequent decomposition reaction is fast and would not allow for XRD detection; additionally, since this would occur only at the graphitic surface, it would not necessarily be detectable by XRD.^208^ Nevertheless, there are several studies in support of the 3D model, with evidence collected by NMR, Raman, EIS, and scanning tunnel microscopy.^208−211^ Moreover, there is indirect evidence for solvent co-intercalation being an important factor in SEI formation. Importantly, it is often the components of the solvation shell that are reduced and form the SEI,^212−214^ and the SEI on the edge sites and basal planes have different chemical compositions.^215,216^ Moreover, Chung et al. presented a study on the *trans* and *cis* isomers of butylene carbonate (BC), showing that Li^+^ reversibly intercalates when using *trans*-BC, while using *cis*-BC leads to exfoliation. Thus, the SEI forming mechanism on graphite is sensitive to the chirality of the solvent. Chung et al. argue that this supports the 3D model as the graphite microstructure would act as a sieve that can discern the two conformers.^208^ Thus, there are simply too many experimental observations of *t*-GICs forming alongside SEI formation to ignore, and it is now the commonly accepted model.^7^ Therefore, a greater understanding of the solvent co-intercalation phenomenon and how to control it might open up new avenues for SEI engineering.

##### 6.1.2.2 The Curious Case of Propylene and Ethylene Carbonate

In a large part, the bad reputation of solvent co-intercalation is due to PC’s destructive reaction with graphite. Curiously, though structurally similar, EC does not cause delamination. Thus, EC and PC provide valuable information on what electrolyte and electrode properties promote or inhibit solvent co-intercalation and should be studied carefully (Figure 14).

First, however, it must be stated that it is not the co-intercalation of PC that causes the delamination of graphite; rather, it is the subsequent reduction of PC that fails to form a blocking film, instead releasing propane gas in the graphite interior that is the direct cause.^14^ Several ways to stop graphite from delaminating in PC-based electrolytes have been identified, including using very high salt concentrations, cycling at lower temperatures, or simply reducing the amount of PC in the electrolyte.^217−225^ The majority of papers argue that the suppression of graphite delamination is due to the formation of a blocking SEI, often with inorganic decomposition products of the anion, which stops the continuous intercalation and reduction of PC. Notably, these strategies also cause the solvation shell to become less stable, thus lowering the desolvation energy barrier.

Although there is plenty of evidence of PC co-intercalate forming *t*-GICs before being reduced, there are only a few papers presenting evidence for EC also doing so.^203,204^ Thus, in essence, there might not be any fundamental difference between EC and PC with regards to co-intercalation; rather, the difference lies in the reduction products, their solubility, and ability to block any further solvents from entering the graphite structure, thus limiting intercalation of solvated ions to the surface of the material.^226^ For instance, the amount of intercalated EC seems to be highly dependent on the type and concentration of linear carbonate it is mixed with, and the surface chemistry of the graphite used, where graphite with few electrochemically active sites fail to gain a blocking surface film.^227^

Thus, the differences seen between EC and PC show the importance of kinetics and surface reactivity in allowing for stable or destructive solvent co-intercalation. By making the desolvation process more sluggish and the surface less reactive, EC can be made to co-intercalate, leading in this case to poor cyclability. In contrast, destabilizing the solvation shell and hence lowering the barrier of the desolvation process, or allowing other solvents/anions known to form blocking SEIs to be a part of the solvation shell, seems to suppress continuous solvent co-intercalation in the case of PC.

Therefore, it can be concluded that for reversible co-intercalation to occur, the electrolyte formulation must yield solvation shells that are (i) energetically stable, such that the desolvation process becomes energetically unfavorable, and (ii) reductively stable, such that no blocking and/or destructive products are formed in neither the bulk nor surface of the graphite. Both PC and EC seem to fulfill the first criteria, yet both fail the second one: PC does not form a blocking film—instead, the reductive products are destructive—while EC decomposes, forming a blocking SEI leading to bare ion intercalation. The importance of properties (i) and (ii) was also concluded in the important paper by Yoon et al.^126^ In addition, a small solvation shell further facilitates solvent co-intercalation as the interlayer distance does not have to expand as much and the layers are in closer proximity to the central positive charge of the solvated ions.^42,86^ In addition, to promote reversible solvent co-intercalation the electrode material should be easy to expand and have low surface and bulk reactivity.^86,203,227^

#### 6.1.3 Diffusion Kinetics and Charge Transfer

Co-intercalation reactions will show notably different charge transfer and diffusion properties compared with conventional ion intercalation. In a favorable situation, co-intercalation may therefore enable reactions with very good rate capability. For example, it has been shown that for intercalation reactions, stripping of the solvation shell is the rate limiting step during the electrochemical process.^228−230^ This is especially observed at low temperatures.^62,80,151,228−231^ Eliminating the desolvation step during charge transfer through solvent co-intercalation may therefore benefit the overall reaction kinetics and hence enable fast charging/discharging capability. Various factors contribute to whether co-intercalation or desolvation takes place during charge transfer. For example, a strong solvation shell aids the co-intercalation process (see sections 3 and 5.1). Ethers generally coordinate quite well to ions, which is why they can show much higher desolvation energies compared to carbonates. For example, DFT calculations showed that the desolvation energy for Na^+^ in 2G (123.5 kJ mol^–1^) is much higher than that for THF (68.7 kJ mol^–1^), EC (59.5 kJ mol^–1^), or DEC (46.8 kJ mol^–1^), enabling the co-intercalation process for 2G.^42^

Moreover, diffusion of solvated ions in the electrode can be very fast and, in some cases, also faster compared with diffusion of the bare ions. GITT measurements are frequently used to determine the diffusion coefficient, and fast diffusion has been confirmed for co-intercalation reactions of Na^+^ and K^+^ in graphite, for example.^22,115^ One explanation for the fast diffusion kinetics in graphite is that the large interlayer caused by the co-intercalation reaction eases diffusion between the stacked graphene layers. In addition, the charge of the intercalated ion is shielded and therefore reduces the Coulomb interaction between the ions and negatively charged graphenes.^86^ For the diffusion of solvated Na^+^ and K^+^ in graphite, apparent diffusion coefficients in the range of 10^–9^ to 10^–8^ cm^2^ s^–1^ were determined.^60^ In more detail, the kinetics at certain SOC values (such as the plateau voltage region) seem to differ between sodiation and desodiation, which is likely connected to a higher amount of solvent being intercalated during sodiation, hampering diffusion.^60^ Other GITT experiments show that the apparent diffusion coefficients are counterintuitively connected with the particle size, showing faster diffusion coefficients for larger particles.^22^ However, the existing knowledge is not sufficient to draw general conclusions, and more systematic studies are needed to better understand the reaction kinetics of co-intercalation reaction as a function of the composition (type of electrode, ion, and solvents), state of charge, and temperature.

### 6.2 Challenges

The concept of solvent co-intercalation also poses some challenges that need to be addressed. Limited capacity and volume expansion can be considered as the most important challenges, which can be limitations for the concept in practice.

#### 6.2.1 Volume Expansion and Mitigation Strategies

**Figure 15:**
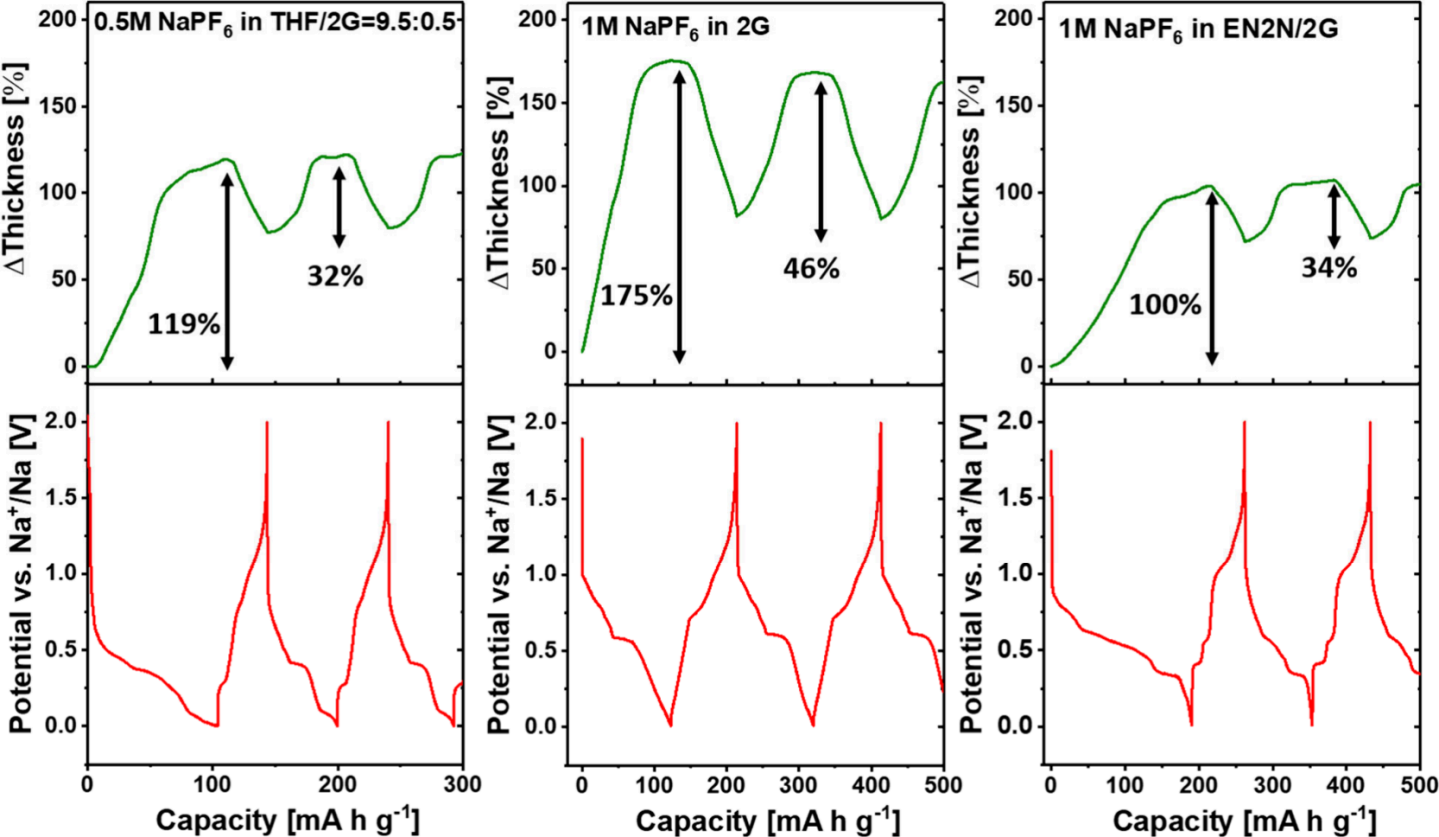
Electrode breathing of co-intercalation reactions for graphite using THF/2G, 2G, and 2G + 10 vol % EN2N/2G as electrolytes. Results are obtained by operando electrochemical dilatometry. The results clearly show the impact of the electrolyte composition on the degree of electrode breathing. Reproduced with permission from ref (41).

The volume expansion of the electrode during co-intercalation has been well studied for the intercalation of solvated Na^+^ in graphite using diglyme as solvent. While the reaction is highly reversible and provides stable and long cycle life, it causes a large electrode expansion/shrinkage (“breathing”) during cycling.^82^ This is typically not a problem in lab cells, although in contained real batteries large volume changes can cause poor cycle life. A strategy for mitigating volume expansion is to optimize the type and amount of binder. Escher et al. compared the use of poly(vinylidene difluoride) (PVDF) and carboxymethyl cellulose (CMC), with the latter showing a less severe breathing.^40^ Another strategy is to alter the electrolyte composition. Thus, Zhang et al. and Escher et al. reported that by using ethylenediamine (EN) as the cosolvent for a 2G-based electrolyte, the expansion of the graphene interlayer space as well as the electrode can be significantly reduced.^39,40^ This approach can be extended to other solvents that are unable to show co-intercalation on their own but show co-intercalation when being added to 2G. Examples are tetrahydrofuran (THF) and 1,3-dioxolan (DOL), which do not show co-intercalation of graphite. Adding a small amount of 2G, however, leads to co-intercalation of these solvents too. The result is quaternary graphite intercalation compounds (q-GICs) that consist of ions, reduced graphite, and two different solvents. This approach as led to a smaller degree of electrode expansion.^41^ A comparison of the electrode expansion variation with different electrolytes is displayed in Figure 15. Note that these mentioned examples refer to graphite as an electrode. The situation may be different when considering cathode materials.

A last approach to mitigate issues related to the volume expansion is mixing the co-intercalation electrode with other materials that show less breathing behavior, e.g., by mixing graphite with hard carbon. While the former shows large breathing, hard carbon electrodes show only a very small degree of breathing , well below 5%.^232^ This composite approach may enable the design of electrodes combining not only capacity but also high rate capability. Also, Sn/graphite composites have been studied.^233^

#### 6.2.2 Limitations in Capacity and Energy Density

One of the main challenges of the co-intercalation approach is the development of full batteries with high energy densities. This is because the capacity of graphite for co-intercalation reactions so far is limited to about 110 mAh g^–1^ and therefore much lower than the 372 mAh g^–1^ that can be reached for the intercalation of Li in graphite.^18,46^ It is of note, however, that the loss in capacity for co-intercalation reactions compared to intercalation is very specific to graphite. Layered cathode materials do not show the same loss in capacity. Moreover, the redox potential for co-intercalation in graphite for Na shows the main redox plateau in the 0.6–0.75 V vs Na^+^/Na range.^51,53^ While this voltage plateau benefits rate performance, it also decreases the energy density compared to electrodes operating closer to 0 V. For example, Li intercalation in graphite occurs mainly around 0.1–0.2 V vs Li^+^/Li.^23,207^

**Figure 16:**
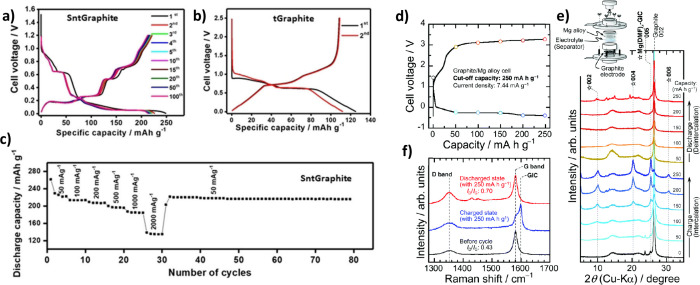
Charge–discharge curves of (a) SntGraphite and (b) tGraphite, measured at 50 mAg^–^1. (c) Rate capability of the SntGraphite electrode measured from 50–2000 mA g^–1^. Measurements were performed in two-electrode geometry with sodium as the counter electrode and 1 M NaPF_6_ in diglyme. Capacity values refer to the weight of SntGraphite. Reproduced with permission from ref (233). (d) Charge and discharge curves for a graphite composite electrode under galvanostatic charge–discharge conditions, (e) XRD patterns, and (f) Raman spectra of the graphite electrode recorded using a two-electrode-type cell in 0.5 M Mg(TFSA)_2_ in DMF. Reproduced with permission from ref (236).

Different strategies have been proposed to overcome the limited capacity challenge. For example, the introduction of other active species that can increase the specific capacity, such as through a conversion reaction, has been reported.^233^ In this sense, tin has been already demonstrated to form a stable electrode with graphite.^233^ The advantage of this strategy is that graphite exhibits a co-intercalation mechanism, while tin forms an alloy compound. Thus, the detrimental volume expansion of the tin compounds can be mitigated due to the expansion of the crystal lattice of the graphite electrodes (see Figure 16a–c). The employment of metal chloride graphite intercalation compounds is another approach to increase the energy density of the battery.^234^ In this regard, Li et al. used MGIC-AlCl_3_ (microcrystal graphite intercalated) electrode structures that provided a specific capacity of 180 mAh g^–1^ stemming from a mixed co-intercalation and conversion reaction of sodium-ions and the host material.^235^ We envisage that this strategy can be important for high-capacity anode materials such as alloys that suffer from a huge volume expansion and therefore poor cyclabilities. Finally, the authors also point out that the role of surface groups has been completely overlooked so far.

The investigation of alternative nongraphite electrodes such as transition metal dichalcogenides is another promising alternative. The recent reports on solvent co-intercalation of sodium with TiS_2_ represent the clearest examples.^42,69,72,237^ Finally, other materials such as the conjugated polymer of poly(*p*-phenylene) (PPP), which has the advantage of delivering a higher specific capacity than that of graphite with glyme-based electrolyte,^79^ Mxenes,^76−78^ and titanates have been explored as alternative co-intercalated electrodes.^80^

Another possibility to increase the capacity would be if smaller solvation shells were to be intercalated such that a larger amount of solvation shells would fit. So far, glymes are the most studied types of solvents, with all showing similar capacities.^8^ Nevertheless, other types of solvents have been reported for solvent co-intercalation, although with a limited capacity.^39,40,63,68^ It has to be mentioned that the introduction of other salts has not modified this parameter up to now.^40,53^

The last strategy to increase the capacity of co-intercalation systems is the use of multivalent cations (Ca^2+^, Mg^2+^) that can carry more charge so that the capacity can significantly increase compare to the analogous alkali compounds (Li^+^, Na^+^, and K^+^).^25,26^ However, the application of multivalent ion insertion is still facing difficulties stemming from sluggish migration of multivalent ions within the electrolyte and electrode. In addition, most of the measurements are performed in a half-cell configuration, which requires the use of an appropriate counter and reference electrode. The most common strategy is to use the corresponding metal as a reservoir of cations. However, the passivation layer formed on this metal electrodes,^110^ in addition to the unreliability of the Ca and Mg as reference electrodes due to the potential shifts and instability,^238^ make the measurements a challenge. Furthermore, even though the mechanism studied in this Review does not have a desolvation step, this step must be considered in half-cell systems with these cations. Thus, the solvation/desolvation kinetics on the counter electrode, in addition to the mass transport limitations on the electrolyte, contribute to a high resistance on the metal counterpart. In spite of that, there have been already reports on the demonstration of co-intercalation with multivalent cations. Kim et al. proved the solvent intercalation of glymes with Mg^2+^ in graphite. Even though the reaction exhibited a very high polarization resistance, it exhibited a constant capacity of 180 mAh g^–1^.^25^ In a similar way, Shimizu et al. showed that DMF can form ternary intercalation compounds with a capacity of approximately 200 mAh g^–1^(see Figure 16d–f).^236^ Ca^2+^ has been also reported to co-intercalated with dimethylacetamide (DMAc) in graphite, as shown by Park et al.^66^ and Yi et al.^65^ In this case, the expansion of the lattice here was significantly smaller than the expansion of the graphite lattice during co-intercalation of Na in ether-based electrolytes.^66^ Further studies from Prakabar et al. from 2019 showed the intercalation of solvated calcium ions in graphite using Ca(TFSI)_2_ in tetraglyme. The reversible capacity is still small (62 mAh g^–1^), and more studies are required.^64^

Additionally, the integration of dichalcogenides with multivalent cations represents an additional step. Tchitchekova et al. discussed in 2018 the intercalation of Ca^2+^ and Mg^2+^ in this dichalcogenide by using a mixture of EC:PC as a solvent. Measurements with XRD suggested a mechanism where the first phase built was established via co-intercalation of the solvents leading to expansion of the lattice along the *c*-axis.^75^ This is in contrast to the findings that no bare Mg^2+^ was intercalated and further insertion of Ca leads to a conversion reaction at the expense of unreacted TiS_2_. These findings were corroborated by Verrelli et al., proving the reversible solvent co-intercalation with PC solvent.^73^ A similar concept was employed in MoS_2_ to co-intercalate magnesium ions.^74^ In this case, the authors selected dimethoxyethane as the solvent to reduce the sluggish kinetics and lower the diffusion barriers with specific capacities of 120 mAh g^–1^ at 0.5 A g^–1^.

#### 6.2.3 The Difficulties of Electrochemically Forming and Detecting Solvent Co-Intercalation Compounds

At this point, the reader might wonder about the limited amount of systems that exhibit a solvent co-intercalation mechanism. However, when evaluating the early works from Whittingham^35^ and Zabel and Solin,^11^ it is clear that more ternary (and quaternary) intercalation compounds have been chemically synthesized. These works are related to graphite and different layered chalcogenides with a variety of different solvents, such as ethers like THF and glymes, aromatic hydrocarbons like toluene, benzene, and furan, amines, amides, and sulfur derivatives such as DMSO.

When analyzing the electrochemical performance of any host, the conventional approach is to measure in a half-cell configuration with the metal counterpart as the cation reservoir. This implies that the metal that works as counter and reference electrodes has to be inert (or have a low reactivity) to the electrolyte. This is the case of ethylenediamine that reacts with the sodium metal to form a characteristic blue electride species and release hydrogen, compromising the stability of the cell.^39^ Zhang et al. and Escher et al. minimized this problem by mixing it with glymes (2G and 4G), which was sufficient to inhibit the chemical reaction and investigate new co-intercalation compounds.^39,40^ A similar problem has been encountered with dimethyl sulfoxide (DMSO), which produces a slow reaction with sodium and a violent reaction (causing the solution to blacken) in the case of potassium.^239^ In both cases, methane evolved as one of the products. We suggest here the use of an alternative counter/reference electrode that exhibits a sufficient capacity to provide ions to the electrochemical reaction and stable and reversible performance during cycling. Thus, it can be possible to access the chemical compounds that have been already reported, although their electrochemical performance has not been studied. There are other ways to solve this problem. For instance, it has been reported that neither THF nor DOL alone have led to the formation of *t*-GIC.^20,53^ However, when those solvents are combined with a glyme solvent, even in very small amounts, ternary and quaternary intercalation compounds can be formed.^41^ Finally, it is worth mentioning that certain solvents might not be active at room temperature, with low capacity values, and hence slight increases of temperature can enable the co-intercalation reaction.^8^

## 7 Solvent Co-Intercalation in Full Cells

**Figure 17:**
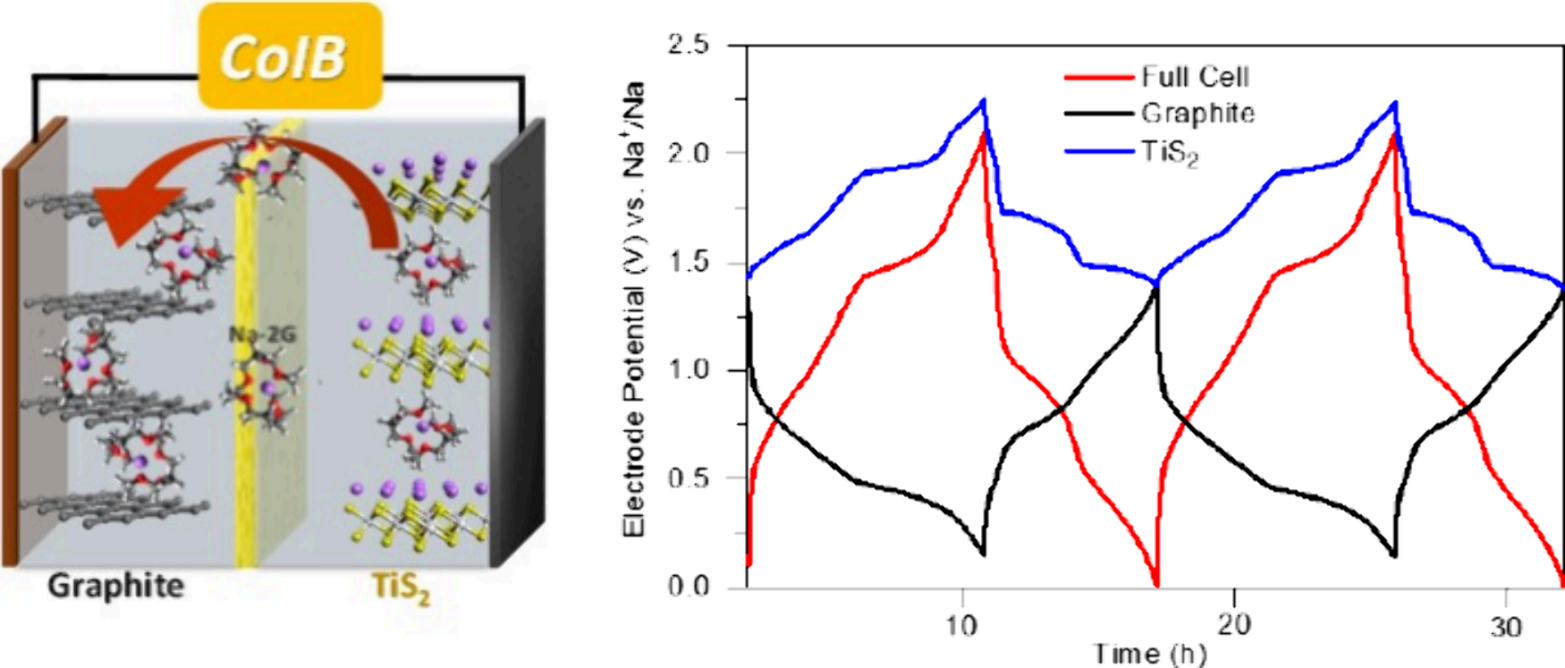
Schematic illustration of a co-intercalation battery (CoIB), i.e., a battery in which both electrodes operate via a co-intercalation process. The example shows a cell with graphite as the anode, TiS_2_ as the cathode, and 1 M NaPF_6_ in 2G as the electrolyte. Reproduced with permission from ref (42).

Evaluating the potential of co-intercalation reactions for practical batteries of hybrid devices requires the assembly of full cells. So far, there have been several reports on full cells using at least one electrode operating via solvent co-intercalation.^20,42,57,84,151,231,240−246^ All studied relied on ethers as solvents and used graphite as the anode, combined with a variety of intercalation cathode active materials (cobalt oxide, sodium vanadium phosphate (NVP), and Prussian white) to create a full cell. In one case, however, the cathode also operated via a co-intercalation reaction. Ferrero et al. assembled a “co-intercalation battery” (CoIB) by pairing pre-sodiated TiS_2_ as cathode with graphite as anode, see Figure 17).^42^

**Figure 18:**
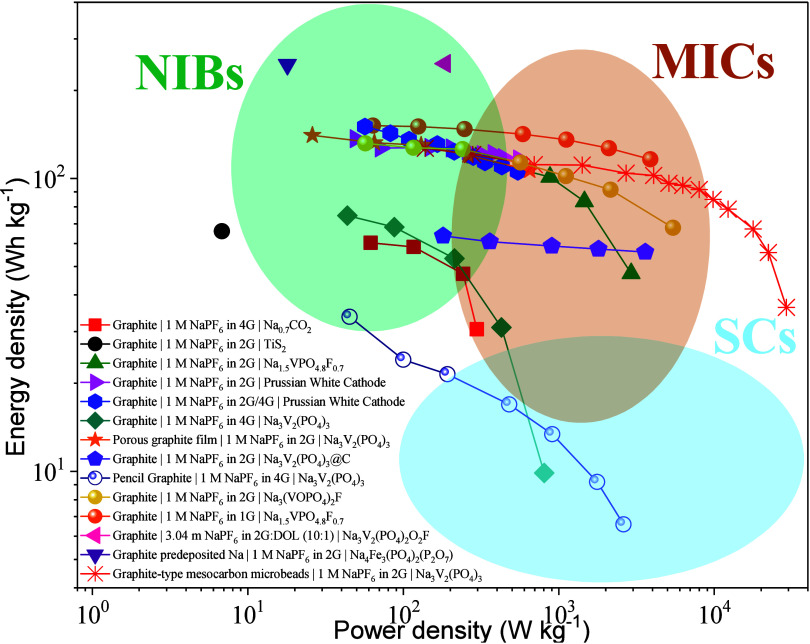
Ragone-like plot with full cells reported on the literature under solvent co-intercalation and comparison with supercapacitors (SCs), sodium-ion batteries (NIBs) and metal-ion capacitors (MICs).^20,42,57,84,231,240−245,251,252^

Figure 18 compares the performance of the full cells with co-intercalated electrodes reported in the literature. Despite the fact that the energy density obtained is still inferior to state of art LIBs and SIBs,^247^ certain cells showed very high power densities. In terms of performance, co-intercalation reactions could therefore help to bridge the gap between batteries and supercapacitors, a field in which metal-ion capacitors and dual-ion capacitors are currently leading the way.^248,249^ One of the shortcomings for these devices is the limited rate capability of the anode material.^249^ Here, co-intercalation could be an approach to overcome these limitations.^233^

In addition, as pointed out by Yang et al, the solvent co-intercalation reaction can find a niche in low-temperature applications.^151^ As mentioned above, stripping of the solvation shell during charge transfer can be the rate-limiting step of the electrode reaction.^21,151,228,231,250^ Although the cell had a lower energy density than a conventional system at room temperature, the authors demonstrated operation at −60 °C with good cyclability.^151^ Regarding low-temperature performance, it is important to note that the solvents commonly showing co-intercalation (ethers) can show a wide liquid range with low freezing points. For example, the melting and boiling points of diglyme are −64 and 160 °C, respectively.

### 7.1 Cathode Materials

As was already shown before and in Figure 18, the majority of the studies on solvent co-intercalation have been focused on graphite electrodes as anodes. However, as the reader has noticed, there is a lack of electrode materials that can be used as cathodes. The ideal material requires a lot of space in the structure that can be easily expanded. However, in this regard, we see that two opposite design criteria might be applied to the cathode and anode. To maximize the full cell voltage, the interlayer binding energy of the anode should be as low as possible, such that the cost of expanding the structure is kept to a minimum.^42^ The cathode, however, should have an interlayer binding energy as close to the maximum binding energy possible that still allows for solvent co-intercalation. Combining these two criteria should lead to full cells with higher potential differences. Even though Ferrero et al. employed TiS_2_ as a sodium reservoir and paired it with graphite to construct the co-intercalation battery (CoIB),^42^ this dichalcogenide material does not represent a true cathode material since it has to be presodiated. One of the reasons for the fewer works of cathode materials on solvent co-intercalation can be ascribed to the rigidness, or the lower ability to be expanded, of the structure during intercalation/deintercalation without compromising its framework. However, we have observed that the co-intercalation behavior might have been overlooked or hidden behind other reactions. That is the case not only in the previous work by Ferrero et al.^42^ but also in the work reported by Sun et al. where they observed for the first time the existence of solvent co-intercalation reactions in a series of layered cathode active materials (e.g., P2-, P3-, or O3-type Ti-, V-, or transition metal mixture-based layered sulfides) when using PC and a 2G solvent electrolyte, unlike conventional carbonate mixtures.^89^ This mechanism, however, is not constant and occurs when the sodium amount in the layered structure is low enough that there is enough space for the solvents to enter, e.g., only layered cathodes with low interlayer binding energy and high interlayer free volume are capable of co-intercalation. Another special case is water intercalation in layered cathode materials, where the layered cathode active material reacting with water is considered as a degradation mechanism of the air-sensitive layered oxides/sulfides when exposed to humid air or water.^253^ However, the air sensitivity of the layered cathode active materials is beyond the scope of this Review, and readers are referred to the article by Yang et al.^254^

A different alternative to obtain cathode materials with these properties is their synthesis in the co-intercalation state. This is the strategy followed by Zong et al., who synthesized co-intercalated Zn^2+^ and C_5_H_14_ON^+^ into a hydrated vanadium oxide.^81^ The structure generated exhibits a higher interlayer distance that provides more active sites. However, it has to be mentioned that the electrochemical process followed here is the intercalation of bare cations (Zn^2+^). The solvent ions are used to strengthen the structure and alleviate the electrostatic interactions between the cations and the host framework. Nevertheless, this could be a promising strategy to follow.

### 7.2 Electrolyte Amount

It is important to emphasize that co-intercalation reactions require a higher amount of electrolyte compared to conventional ion intercalation reactions. Depending on how many solvents are being co-intercalated/deintercalated, one needs to account for this additional electrolyte when calculating energy and power densities. This is very similar to devices such as supercapacitors or dual-ion batteries, where an “extra electrolyte” is also required. However, since the amount of co-intercalating solvents is still not clear for many co-intercalation reactions, a fair evaluation is difficult so far. To date, the energy and power densities displayed on the reports (see Figure 18) only account for the total active mass of the anode and cathode. A more detailed analysis of the achievable energy and power densities should therefore be an important task for the future.

**Figure 19:**
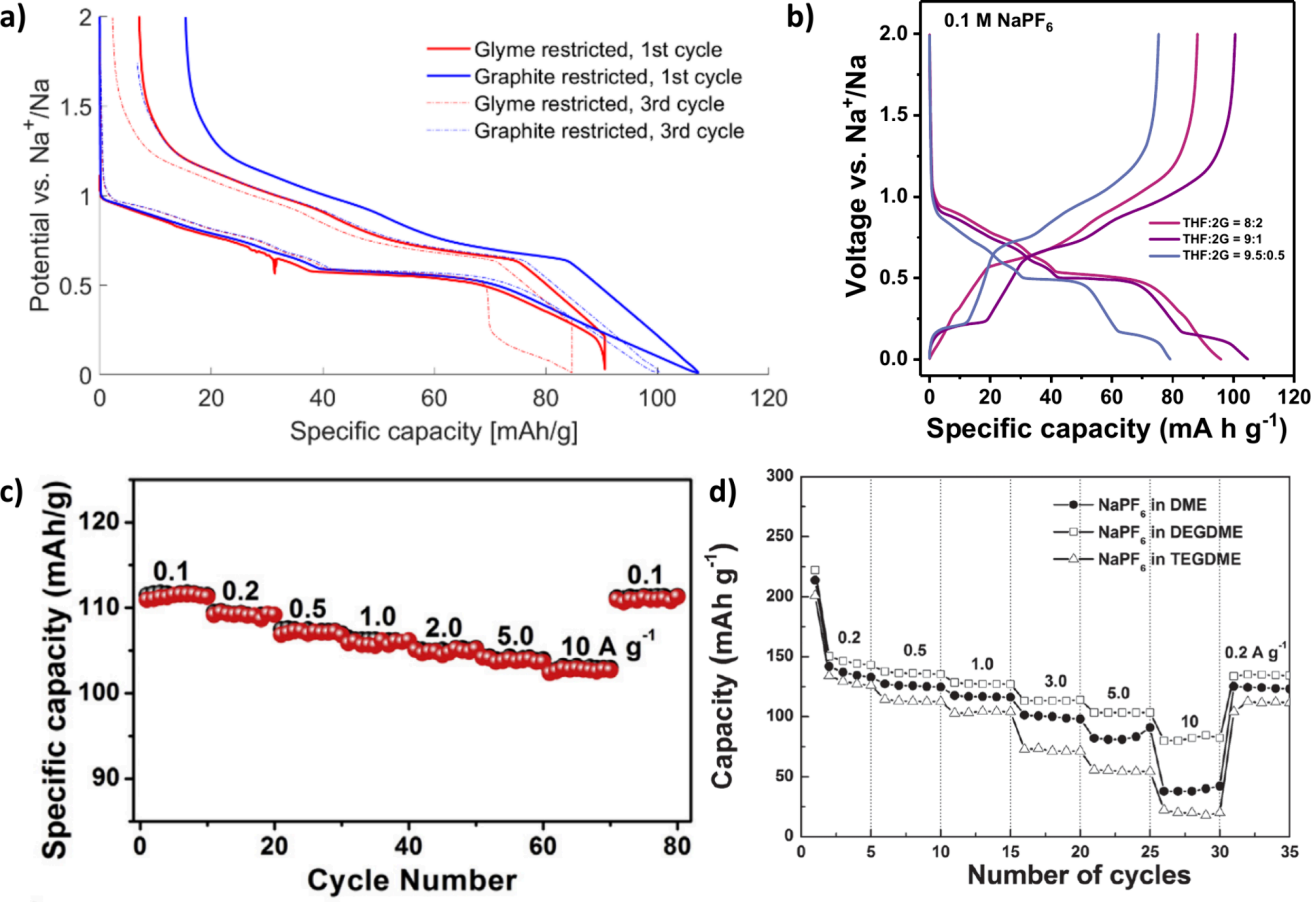
(a) Example of two cells where the normal case (blue) shows a setup in which the graphite is limiting the reaction and full sodiation is achieved; once there are too few G1 molecules in the cell to fully sodiate the graphite, an abrupt stop in sodiation is seen (red). Reproduced with permission from ref (86). (b) Change of voltage profile by different ratios of THF/2G solvent at 0.1 M in same salt concentration. Reproduced with permission from ref (41). Rate capability test performed with graphite as the electrode material (c) without and in tetraglyme^57^ and (d) with addition of conductive carbon in various electrolytes.^20^

In the lab scale, more electrolyte is used for experiments, making the active material the limiting reagent in the electrochemical reaction, especially in half cells. Recently, Åvall et al. showed that using a lower amount of glymes than required can lead to an incomplete reaction, resulting in a lower capacity and a sudden drop in voltage (see Figure 19a).^86^ It has to be mentioned that since the amount of solvents bound to a specific cation might differ between each other, the optimal amount of electrolyte can also be changed. For example, Son et al. explored the difference in the electrolyte solvents with respect to the Na and graphite electrode.^41^ Since this system was composed of two solvents (2G and THF), the modification of the ratios can lead to change on the voltage profile, with in this case a lowering of the main voltage plateau and the appearance of a second plateau at higher states of sodiation (see Figure 19b).^41^ However, the use of diglyme was restricted as an additive so that a small amount was sufficient to change the mechanism. Therefore, the optimization of the ratios of active material/solvent/ion has to be done independently for each system, and more investigations, especially in full cells, are needed.

### 7.3 Practical considerations

Separators are porous membranes that physically separate the cathode and the anode and allow ionic transport during the electrochemical process. They should provide in addition good and stable mechanical, thermal, and electrochemical properties during operation. Most of the publications about this topic used glass fiber (Whatman) as the separator,^8,40,42,51,55,58,82,83,86,93^ while in most commercialized LIBs polyethylene and polypropylene have been used as separators.^255^ However, this polyolefin separators suffer from low wettability, which is fundamental for an application that depends at a higher extent on the electrolyte solvent mobility, such as the one we are discussing on this work.^255,256^ On the other hand, since the porosity is lower than that of the glass fiber separators, they might require less solvent electrolyte. This is of particular interest since the solvent participates in the reaction and there is a risk of wettability decrease on the separator (when using low amounts), especially during the charging process, that can make the reaction incomplete. Thus, a less porous separator that can maintain the mechanical and electrochemical properties will be beneficial, not only to limit the amount of solvent employed but also as a way to increase the gravimetric energy density of the device.

In the case of additives, conductive carbons (such as Super-P or Carbon black) are normally used to improve the performance of the electrode material in respect to the electrical conductivity and the ionic conductivity.^257^ Graphite, as an electrode material for the co-intercalation process, exhibits a high conductivity and is already displaying a very good rate capability; thus, it might not encounter problems with respect to these properties. There is no specific study on the effect of conductive carbon on the performance of graphite for this process. Nevertheless, results of Kim et al. and Wang et al. showed an increased in the specific capacity and an inferior rate capability compared to the graphite without any additives.^19,20,57,109^ Since the addition of conductive carbon lowers the rate capability and does not add any real specific capacity to the graphite electrode, the usage of Super-P in this special case might be counterproductive (see Figure 19c and d). Conductive carbons have been used with other materials such as dichalcogenides to improve the electrical conductivity of the electrode.^42^ In this case, the authors ascribed the tail-like feature at low voltages for different electrolytes, on the first cycle, to the presence of a conductive carbon additive, since it was not observed with the pure active material. Therefore, these irreversible processes were linked to the carbon additive, which is the result of a lower ICE compared to the pristine material. Nevertheless, the improvement of conductive properties while maintaining a low ICE might be an interesting aspect of future research work.

Overall, there is a lot of research left to be carried out on full cells employing the solvent co-intercalation reaction. To the best of the authors’ knowledge, there are no studies on the effect of calendaring or pressure carried out so far and very few on particle size and morphology,^22^ and the community has not taken the next step in cell assembly: a single-layer pouch cell.

## 8 Summary and Outlook

This Review discussed various key characteristics, advantages, and challenges of co-intercalation reactions in electrode materials. While formerly considered as a highly detrimental process leading to rapid degradation of the electrode material, recent results indicate that redox reactions involving solvent co-intercalation can be highly reversible. Most importantly, there is a great wealth in possible co-intercalation compounds, thanks to a large number of combinations between intercalating ions, solvents, and host structures. While the co-intercalation reaction of Na^+^ with diglyme as the solvent and graphite as the host can be considered as reference system, many more reactions involving other ions, solvents, mixtures of solvents, or other layered host structures have been reported. The concept therefore provides a very versatile handle to tune the properties of electrodes but also to explore new so-far unknown compounds.

While compounds with co-intercalated solvents can also be synthesized via chemical methods (which was a very active research field in the 1970s and 1980s for graphite as the host material), electrochemical synthesis provides some unique advantages. These include, for example, the fact that thermodynamic data (e.g., redox potential and entropy) can be obtained, that the stoichiometry of the materials (capacity) can be easily adjusted, and that the reversibility of the reaction can be easily studied (cell cycling).

A wealth of questions arises when considering co-intercalation reactions. Understanding the charge transfer process and the nature of the “SEI-free” interface, understanding the limits of the reaction in terms of capacity and redox potentials, and exploring the number of combinations (ions, host structures, solvents) enabling co-intercalation appear as the most urgent questions. While the knowledge has significantly increased in the past few years, many aspects of the reaction remain unchartered territory. Understanding the specific interactions between host structure, solvents, and ions using theory and advanced characterization tools will be critical for optimizing the redox potential, capacity, and diffusion properties. Theoretical studies have already been used, and some important metrics have been identified that point toward a solvent co-intercalation reaction occurring (for a given electrode/electrolyte combination). However, the different simulations depend heavily on the initial configuration proposed by the researcher, which is challenging in the case of solvent co-intercalation where this knowledge is often lacking. In addition, some studies are focused on the properties of non-realistic conditions (such as electrolyte in isolation), which is insufficient since the interaction between the electrolyte and the host electrode is fundamental.

Future studies should also address whether the concept is viable from a practical perspective. To increase energy density, the amount of co-intercalating solvent molecules should be minimized, and the cell voltage and electrode capacities should be maximized. It is clear, however, that the potential benefit of co-intercalation reactions lies in the high-rate performance thanks to the possible fast diffusion and minimized charge transfer resistance, as well as the long cycle life. Another challenge is the large volume expansion of the reaction, which can cause problems in real devices. Co-intercalation of specific solvents or pillaring of the structure may help to mitigate these issues. On the other hand, the high reversibility of some of the co-intercalation reactions along with their large and reversible volume expansion may also be attractive in view of developing electrochemical actuators or switches.

More recently, the formation mechanism of co-intercalation compounds has been found to have an additional level of chemical complexity, as shown for graphite^86^ and Na_*x*_TiS_2._^89^ For graphite, it was found that when sodiation starts, not only solvents bound to Na^+^ but also free solvents enter the structure that diffuse freely. Because of the facts that (a) free solvents intercalate and (b) the graphene interlayer expands to values easily exceeding 1–2 nm (which correspond to the micropore and mesopore regimes),^112^ the co-intercalation process can also be considered as pore filling.^86^ This means that plenty of different solvents may participate in co-intercalation reactions. Since the co-intercalation reaction with graphite can be highly reversible, easily reaching several thousand cycles, the process can be interpreted as making graphite a switchable porous material.

This peculiar behavior of co-intercalation reactions also shows some overlap with other research fields. The interaction of solvated ions with porous materials is also highly relevant to the development of supercapacitors, where the boundaries between Faradaic and non-Faradaic charge storage become blurred. It is also of note that graphite, the most discussed host material in this Review, can store not only cations (Li^+^, Na^+^, etc.) but also anions (PF_6_^–^, TFSI^–^, or [AlCl_4_]^−^).^258−261^ Anion intercalation occurs at high potentials and is explored for dual-ion batteries and Al-ion batteries, for example. An accompanying intercalation of solvents has not been reported for such cases but seems to be not unreasonable. The intercalation of [AlCl_4_]^−^ is conceptually closest to solvent co-intercalation reactions since the chloride anions shield the charge of the cation, similar to [Na(diglyme)_2_]^+^, for example. Interestingly, the capacity of graphite for the different anionic intercalants (80–150 mAh g^–1^) is close to the cases of solvent co-intercalation.^260,262−264^ Lastly, one has to realize that there might be an attractive link to catalysis in nanoconfined spaces. The co-intercalation of free solvents (that could be possible reactants) between the graphene interlayer space, along with the fast diffusion and adjustable electrode potential, may therefore also open up opportunities for (electro)catalysis under nanoconfinement.^32,265^
